# Systematic review and meta-analysis of randomized controlled trials evaluating the efficacy of non-surgical periodontal treatment in patients with concurrent systemic conditions

**DOI:** 10.1007/s00784-023-05392-6

**Published:** 2023-12-26

**Authors:** Prabhakar Joseph, Priya Prabhakar, Birte Holtfreter, Christiane Pink, Jeanie Suvan, Thomas Kocher, Vinay Pitchika

**Affiliations:** 1 Department of Restorative Dentistry, Periodontology, Endodontology, Preventive and Pediatric Dentistry, University Medicine Greifswald, Fleischmannstr. 42, 17475 Greifswald, Germany; 2 Department of Orthodontics, University Medicine Greifswald, Greifswald, Germany; 3 Periodontology Unit, UCL Eastman Dental Institute and Hospital, University College London, London, UK; 4 Department of Oral Diagnostics, Digital Health and Health Services Research, Charité-Universitätsmedizin Berlin, Berlin, Germany

**Keywords:** Metanalysis, Non-surgical periodontal therapy, Periodontal medicine, RCT

## Abstract

**Objective:**

To conduct a systematic review of the published scientific evidence to evaluate the efficacy of nonsurgical periodontal therapy (NSPT) in treating periodontitis in patients with concurrent systemic conditions (diabetes, CVD, erectile dysfunction, chronic kidney disease, rheumatoid arthritis, polycystic ovarian syndrome, obesity, pregnancy). We hypothesised that NSPT results in better periodontal outcomes when compared to untreated controls after follow-up.

**Materials and methods:**

A systematic search (PUBMED/EMBASE) was conducted from 1995 to 2023 to identify randomised controlled trials (RCTs) with a minimum follow-up of 3 months. The primary outcome was the difference in mean probing depth (PD), and the secondary outcomes were mean clinical attachment loss (CAL), percentage of sites with PD ≤ 3 mm (%PD ≤ 3 mm) and percentage of sites with bleeding on probing (%BOP) between the treated and untreated control group in patients with comorbidities.

**Results:**

The electronic search resulted in 2,403 hits. After removing duplicates, 1,565 titles and abstracts were screened according to the eligibility criteria, resulting in 126 articles for full-text screening. Following this, 44 studies were analysed. Restricting to studies with low bias or some concerns, NSPT group demonstrated a 0.55 mm lower mean PD (95%CI: −0.69; −0.41) after 3 months compared to the control group.

**Conclusion:**

Compared to the untreated controls, NSPT notably reduced mean PD, mean CAL, and %BOP while increasing %PD ≤ 3 mm in patients with concurrent systemic conditions. These findings suggest that NSPT is also an effective procedure in managing periodontitis in patients with concurrent systemic conditions.

**Trial registration:**

This systematic review was registered under the protocol registration number CRD42021241517/PROSPERO.

**Supplementary Information:**

The online version contains supplementary material available at 10.1007/s00784-023-05392-6.

## Introduction

The first and second steps in periodontitis treatment are performed non-surgically (NSPT) combined with oral hygiene instructions [1]. Typically, this results in the reduction of probing depth, gain in clinical attachment, resolution of inflammation and arrests the progression of periodontitis [2–4].

Although NSPT is the gold standard for periodontal treatment [5], there is a paucity of randomized clinical trials (RCTs), specifically designed to assess the efficacy of NSPT versus no treatment in individuals with comorbidities [2, 6]. Several authors have argued that a wealth of literature has demonstrated efficacy in other contexts, and a negative conclusion would be unfair [2]. In the last decade, health policy stakeholders have asked for reliable, evidence-based data to recommend or include NSPT in insurance or reimbursement schemes. The Canadian Agency for Drugs and Technologies in Health [7] concluded that NSPT improved periodontal outcomes in adult patients with varying severity of periodontitis with or without systemic diseases but not in patients with incipient periodontitis within a three-month observation period. Based on a thorough literature review, the German Institute for Quality and Efficiency in Healthcare deemed NSPT as a procedure with an uncertain benefit [8].

Since the 1990s, multiple epidemiological, experimental, and interventional studies have revealed that periodontitis may impact systemic health [9]. Through the emergence of “periodontal medicine”, the periodontal community has become involved in the medical field and aimed to demonstrate the impact of periodontal treatment on other chronic inflammatory medical conditions [10]. To substantiate their hypotheses, the periodontal community had to perform state-of-the-art medical experiments with an untreated, periodontally diseased control arm. To overcome the ethical dilemma, the periodontal treatment of the control arm was delayed, until the medical question, e.g., change in biomarker levels, was expected to be answered. Because of a presumptive progression of periodontitis due to non-treatment, the periodontal community restricted the length of delayed treatment to only 3 months in most cases. Few RCTs were performed longer than 6 months [11, 12]. Although these studies were designed to answer different research questions, they can be used to answer the question of NSPT’s efficacy.

This introduction highlights that health policy around the globe asks whether NSPT improves periodontal parameters better than no treatment. Due to the emergence of periodontal medicine, many RCTs that compared immediate and delayed NSPT are available to address the question: "Does NSPT enhance periodontal parameters in patients with periodontitis compared to delayed treatment in individuals with concurrent systemic conditions?”. Even if many RCTs are available, the limitation is that the information stems from patients with concurrent systemic conditions and not healthy individuals. This study aimed to perform a meta-analysis of relevant “periodontal medicine” literature to evaluate the efficacy of NSPT compared to no treatment, supragingival scaling (SGS) or oral hygiene instruction (OHI) (control group) in periodontitis patients with concurrent systemic conditions.

## Methods

### Standards of reporting

The protocol of this systematic review is in accordance with the “Preferred Reporting Items for Meta-Analysis (PRISMA) statement” [13] and is intended to address the following question: "Does NSPT enhance periodontal parameters in patients with periodontitis in comparison to no treatment (no Tx), supragingival scaling (SGS), or oral hygiene instructions (OHI) in individuals with concurrent systemic disease or condition (comorbidity)?" (Protocol registration: CRD42021241517/PROSPERO) [14].

### Eligibility criteria

Studies fulfilling the following criteria were eligible for inclusion: 1) RCTs involving human subjects from 18 years onward, suffering from periodontitis associated with a systemic disease or condition; 2) studies which used NSPT as monotherapy without local or systemic antibiotics, without other physical adjunctive interventions or without periodontal surgery; 3) studies with no treatment, OHI or SGS as control; 4) studies reporting mean PD with a minimum of 3 months post-treatment follow-up. Articles published in languages other than English were excluded.

### Source of information and search strategy

Keywords and MeSH terms were selected, and electronic search strategies were developed for PubMed and Embase (Appendix Table 1). A literature search was also conducted using keywords on Google Scholar. Additionally, a manual search of the references from the included studies was performed. The publications were collected and organised using a reference manager (EndNote X7, Thomson Reuters) and duplicates were excluded. Two reviewers (PJ and VP) independently searched studies published between 01.01.1995 and 30.09.2023.

### Selection process

The study selection process was done independently by two reviewers (PJ and VP) in two phases. Phase-1: two reviewers screened the titles and abstracts of all identified reports, based on the inclusion/exclusion criteria. Phase-2: the full texts of the selected studies were evaluated according to the eligibility criteria. In case of disagreements, a consensus was reached by consulting the third reviewer (TK). Excel spreadsheets were used to record the decisions (Appendix Table 2).

### Data collection process and data items

Two reviewers (PJ and PP) independently extracted relevant data from the included studies, such as study population, interventions, comparisons, reported outcomes, baseline and follow-up values and conclusions. This information was filled in Excel spreadsheets to provide an overview of the available data. Discrepancies were solved by consensus discussion with VP and TK, and the values were updated.

The primary outcome was mean PD. Mean clinical attachment loss (CAL) in mm, percentage of sites with bleeding on probing (%BOP) and percentage of sites with PD ≤ 3 mm (%PD ≤ 3 mm) were the secondary outcomes assessed in this review. For all outcomes, means and standard deviations were extracted at 3 and 6 months for NSPT and control groups (Appendix Table 3).

### Risk of bias assessment

Two reviewers (PJ, VP) independently assessed risk of bias for included RCTs, according to the Cochrane Collaboration risk-of-bias tool for randomized trials (RoB2) [15]. Each study was graded according to five domains: randomization (D1), deviation (D2), missing data (D3), outcome measurement (D4) and selective reporting (D5), and an overall score for risk of bias was assigned. Discrepancies raised were discussed with two researchers (TK and BH) until an agreement was reached.

### Effect measures and synthesis methods

The mean difference and the 95% confidence interval (95% C.I.) for all outcomes between the test and the control arm at the 3- and/or 6-month follow-ups were calculated. Negative estimates favour the NSPT group over the control group except for %PD ≤ 3 mm. Studies with a low risk of bias or rated as some concerns were grouped together and compared to studies with high risk of bias when computing pooled estimates or when plotting forest plots. When median values were reported, they were converted into mean values, and the missing standard deviations were imputed using the average standard deviation from the available studies as prescribed by The Cochrane Collaboration [16].

We performed subgroup analyses in patients with comorbidities, such as, type 2 diabetes, cardiovascular diseases (CVD), erectile dysfunction, pregnancy, and rheumatoid arthritis (RA). Systemic diseases/conditions (obesity, polycystic ovarian syndrome (PCOS), chronic obstructive pulmonary disease (COPD), and chronic kidney disease (CKD)) with less than two studies were excluded from subgroup analyses. To examine the efficacy of NSPT, we calculated the change in means between the pre- and post-treatment values within the NSPT group for the abovementioned variables. Heterogeneity was quantified using the I^2^ statistic, and the publication bias was tested using Egger’s test (Egger et al., 1997) and illustrated outcomes through funnel plots. Random-effects meta-regression was performed by modelling the pre- and post-treatment values of all outcomes on the type of comorbidity, risk of bias, and year of publication.

## Results

### Study selection

The initial search yielded 2,403 articles. After removing duplicates, 1,565 records were screened by title and abstract and 126 full-text articles were assessed for eligibility. A total of 44 studies met the inclusion criteria (Appendix Fig. 1). These studies were published between 1995 and 2023.

### Study characteristics

#### Methodology

All but four trials were single-centre trials. Of the included trials, 25 had a 3-month follow-up period, 20 included a 6-month follow-up, two had a 12-month follow-up, and one had a 24-month follow-up. The 44 trials were performed in different countries as follows: Australia (1), Austria (1), Brazil (8), Chile (1), China (8), Egypt (1), Greece (1), India (7), Iran (2), Japan (1), Jordan (1), Malaysia (2), Pakistan (1), Spain (1), Turkey (2), the United Kingdom (2), the USA (3), and Vietnam (2).

#### Participant characteristics

Overall, 44 studies (3382 patients, ages ranging from 22 to 68 years) were included in the meta-analysis. All patients had periodontitis, although the severity varied. Of the included trials, 21 were conducted in patients with diabetes, 9 in patients with CVD, 6 in pregnant women, 2 in men with ED, 1 in patients with CKD, 2 in patients with RA, 1 in patients with COPD 1 in women with PCOS, and 1 in obese patients.

#### Periodontal interventions and measures

In 26 RCTs, NSPT was performed with curettes and/or ultrasonic instruments, and 19 RCTs did not report the instruments used. NSPT was performed in one to five sessions; however, 15 trials did not report the number of sessions. In the NSPT group, five studies used a chlorhexidine mouth rinse as an adjunct. In the control arm, 21 studies reported no treatment, nine used SGS, and 15 used OHI. In 37 RCTs, periodontal outcomes were measured at four or six sites per tooth. Further information about teeth examined, probe used, and study conclusions are summarized in Table 1.

**Table 1:** Characteristics of all the included studies regarding the sample, systemic disease/condition, periodontal recordings, assessment intervals and study conclusions

Author (Year) Country	N patients (M,F) Age, mean ± SD Smokers, (n)	Systemic disease/ condition	Participants at baseline/dropouts; Age in years, mean ± SD;		Probe; Recording protocol; Recorded sites	Assessment intervals	Conclusions
			Intervention; Instrumentation	Control			
Intention-to-treat criteria							
Adegboye [17] Brazil	33 (0,33) NR 4	Pregnancy	NSPT; 15/6 26.5 ± 6.3 NR	OHI; 18/2 28.8 ± 4.3	NCP 15 Pr FM 6 spt	BL 6 m	No significant difference in mean PD and mean CAL between treatment and control group at 6 m
Caneiro-Queija [18] Spain	40 (0,40) NR Excluded	Pregnancy	NSPT; 20/0 32.14 ± 4.2 HI	SGS; 20/0 32.25 ± 4.2	UNC 15 Pr FM excl 3 M 6 spt	BL 3 m	Significant difference in %BOP and mean CAL between treatment and control group at 3 m
Das [19] India	34 (21,13) NR Excluded	DM	NSPT; 17/0 38 ± 11 NR	No Tx; 17/0 40 ± 12	NR NR NR	BL 3 m	Significant difference in mean PD and mean CAL between treatment and control group at 3 m
El-Makaky [20] Egypt	140 (140,0) NR Excluded	ED	NSPT; 70/0 35.77 ± 2.8 HI & US	No Tx; 70/0 35.71 ± 2.7	Unknown Pr FM excl 3 M 6 spt	BL 3 m	Significant difference in %BOP, mean PD and mean CAL between treatment and control group at 3 m
Eltas [21] Turkey	120 (120,0) NR NR	ED	NSPT; 60/0 38.1 ± 6.9 HI & US	No Tx; 60/0 36.6 ± 6.1	Williams Pr FM excl 3 M 6 spt	BL 1 m 3 m	Significant difference in %BOP, mean PD and mean CAL between treatment and control group at 3 m
Fiorini [22] Brazil	57 (0,57) NR 16	Pregnancy	NSPT; 27/0 NR NR	SGS; 30/0 NR	NC Pr 15 FM excl 3 M 6 spt	BL 6 m	Significant difference in %BOP between treatment and control group at 6 m
Ide [23] United Kingdom	39 (23,16) 47.1 ± 7.0 Excluded	CVD	NSPT; 24/0 47.8 ± 7.5 HI & US	No Tx; 15/0 46.0 ± 6.2	Yeaple Pr FM 4 spt	BL 1.5 m 3 m	Significant difference in %BOP between treatment and control group at 3 m
Kamil [24] Jordan	36 (20,16) NR Excluded	CRP & Chol	NSPT; 18/0 46.7 ± 3.4 NR	OHI; 18/0 45.4 ± 3.3	Williams Pr FM excl 3 M 6 spt	BL 3 m	Significant difference in %PD ≤ 3 mm in treatment group whereas not significant in control group between baseline and 3 m
Kaur [25] India	100 (48,52) NR Excluded	DM	NSPT; 50/5 51.82 ± 5.8 HI & US	No Tx; 50/4 52.94 ± 6.0	Williams Pr FM excl 3 M 6 spt	BL 3 m 6 m	Significant difference in %BOP, mean PD and mean CAL between treatment and control group at 3 m and 6 m
Kiran [26] Turkey	44 (18,26) 54.39 ± 11.7 7	DM	NSPT; 22/0 55.95 ± 11.2 NR	No Tx; 22/0 52.82 ± 12.2	Williams Pr FM 4 spt	BL 1 m 3 m	Significant difference in %BOP and mean PD between treatment and control group, whereas no significant difference in mean CAL at 3 m
Kolte [27] India	60 (35,25) NR Excluded	DM	NSPT; 30/0 NR NR	SGS; 30/0 NR	UNC 15 Pr NR NR	BL 3 m	Significant difference in mean PD and mean CAL between treatment and control group, whereas no significant difference in %BOP at 3 m
Koromantzos [28] Greece	60 (33,27) 59.52 ± 8.88 11	DM	NSPT; 30/4 59.62 ± 7.9 HI & US	SGS; 30/3 59.42 ± 9.8	Unknown Pr FM 6 spt	BL 1 m 3 m 6 m	Significant difference in %BOP between treatment and control group at 6 m
Masi [29] United Kingdom	51 (25,26) NR 2	DM	NSPT; 27/0 56 ± 9 NR	SGS; 24/0 58 ± 11	Unknown Pr NR 6 spt	BL 2 m 6 m	Significant difference in %BOP and no significant difference in mean PD between treatment and control group at 6 m
Milanesi [30] Brazil	158 (77,81) NR 33	DM	NSPT; 79/1 NR HI & US	No Tx; 79/6 NR	NCP 15 Pr FM 6 spt	BL 3 m 6 m	Significant difference in %BOP and mean PD between treatment and control group at 3 m and 6 m, whereas significant difference in mean CAL only at 6 m
Moeintaghavi [31] Iran	40 (20,20) 50.29 ± 3 NR	DM	NSPT; 20/0 NR HI & US	No Tx; 20/0 NR	Williams Pr NR NR	BL 3 m	Significant difference in mean PD and mean CAL between treatment and control group at 3 m
Pinho [32] Brazil	30 (NR) 50 NR	RA	NSPT; 15/0 NR NR	No Tx; 15/0 NR	Florida Pr NR 6 spt	BL 3 m 6 m	Significant difference in mean PD between treatment and control group at 3 m and 6 m; for %BOP only at 6 m
Ribeiro [33] Brazil	42 (NR) NR Excluded	RA	NSPT; 26/0 51.6 ± 10.3 NR	SGS; 16/0 47.7 ± 9.5	NR FM 6 spt	BL 3 m	Significant difference in mean BOP between treatment and control group at 3 m
Sadatmansouri [34] Iran	30 (0,30) NR NR	Pregnancy	NSPT; 24/0 29.1 ± 4.3 HI & US	No Tx; 15/0 28.4 ± 4.1	Williams Pr FM excl 3 M 6 spt	BL 3 m	Significant difference in %BOP, mean PD and mean CAL between treatment and control group at 3 m
Seinost [35] Austria	59 (49,10) NR 26	CVD	NSPT; 29/2 59.6 ± 8.5 NR	No Tx; 30/1 59 ± 8.4	Florida Pr FM 6 spt	BL 3 m	Significant difference in %BOP, mean PD and mean CAL between treatment and control group at 3 m
Singh [36] India	30 (NR) NR NR	DM	NSPT; 15/0 NR NR	No Tx; 15/0 NR	Williams Pr NR NR	BL 1 m 3 m	Significant difference in mean PD and mean CAL in treatment group whereas not significant in control group between baseline and 3 m
Telgi [37] India	40 (NR) NR Excluded	DM	NSPT; 20/0 NR NR	OHI; 20/0 NR	UNC 15 Pr FM 6 spt	BL 3 m	No significant difference in mean PD between treatment and control group at 3 m
Tran [38] Vietnam	64 (28,36) 63.25 ± 8.86 Excluded	DM	NSPT; 32/0 64.63 ± 8.64 HI & US	OHI; 32/0 61.94 ± 9.0	NR NR 6 spt	BL 3 m 6 m	Significant difference in mean PD and mean CAL between treatment and control group at 3 m and 6 m
Vidal [39] Brazil	22 (11,11) NR 4	CVD	NSPT; 11/0 48.9 ± 3.9 HI & Sonic	No Tx; 11/0 49.7 ± 6.0	Hawe “Click Pr” FM 6 spt	BL 3 m	Significant difference in mean PD and mean CAL in treatment group whereas not significant in control group between baseline and 3 m
Wang Y [40] China	58 (33,25) NR 7	DM	NSPT; 29/2 64.4 ± 9.3 HI & US	OHI; 29/1 64.7 ± 8.3	UNC 15 Pr FM excl 3 M 6 spt	BL 3 m	Significant difference in %BOP, mean PD and mean CAL between treatment and control group at 3 m
Zhou S [41] China	75 (60,15) NR 20	CVD	NSPT; 40/0 62.11 ± 9.3 HI & US	OHI; 35/0 62.48 ± 12.2	Williams Pr NR NR	BL 3 m	Significant difference in mean PD and mean CAL between treatment and control group at 3 m
Zhou X [11] China	40 (32,8) NR 10	COPD	NSPT; 20/0 63.9 ± 9.4 HI & US	No Tx; 20/0 68.0 ± 7.6	Williams Pr FM excl 3 M 6 spt	BL 6 m 12 m 24 m	Significant difference in mean PD and mean CAL between treatment and control group at 6 m
Per-protocol criteria							
Akram [42] Malaysia	62 (17,45) NR 11	Obesity	NSPT + CHX; 33/2 44.68 ± 10.6 HI & US	No Tx; 33/2 44.84 ± 9.0	UNC 15 Pr NR 6 spt	BL 1.5 m 3 m	No significant difference in mean PD and mean CAL between treatment and control group at 3 m
Chen [43] China	84 (43,41) NR 17	DM	NSPT; 45/2 57.91 ± 11.3 HI & US	No Tx; 44/3 63.2 ± 8.5	Williams Pr FM excl 3 M 6 spt	BL 1.5 m 3 m 6 m	Significant difference in %BOP and mean CAL between treatment and control group at 3 m and 6 m, Significant difference in mean PD at 6 m only
Deepti [44] India	51 (0,51) 23.50 ± 4.2 Excluded	PCOS	NSPT; 30/4 24 ± 4.3 HI & US	OHI; 30/5 22.6 ± 2.8	PCP-UNC Pr NR 6 spt	BL 3 m 6 m	Significant difference in %BOP, mean PD and mean CAL between treatment and control group at 3 m and 6 m
Engebretson [45] USA	474(NR) NR 66*	DM	NSPT + CHX; 257/17 56.7 ± 10.5* HI & US	No Tx; 257/22 57.9 ± 9.6*	NR FM 6 spt	BL 3 m 6 m	Significant difference in %BOP, mean PD and mean CAL between treatment and control group at 3 m and 6 m
Fang [46] China	97 (55,42) NR 11	CKD	NSPT; 50/2 53.71 ± 5.8 HI & US	No Tx; 50/1 55.53 ± 6.7	Williams Pr FM excl 3 M 6 spt	BL 6 w 3 m 6 m	Significant difference in %BOP and mean PD between treatment and control group at 3 m and 6 m
Hada [47] India	55 (39,16) 60.3 Excluded	CVD	NSPT; 35/5 60.43 ± 10.3 HI & US	No Tx; 35/10 60.21 ± 9.2	Williams Pr FM 4 spt	BL 6 m	Significant difference in %BOP between treatment and control group at 6 m
Kapellas [48] Australia	169 (107, 62) 40.2 ± 10.4 102	CVD	NSPT; 138/51 NR HI & US	No Tx; 135/53 NR	NR FM excl 3 M 4 spt	BL 3 m 12 m	Significant difference in mean PD between treatment and control group at 3 m
López [49] Chile	351 (0,351) 27.56 ± 4.3 NR	Pregnancy	NSPT; 200/37 28 ± 4.5 NR	No Tx; 200/12 27 ± 4.3	Unknown Pr FM excl 3 M 6 spt	BL 3 m	Significant difference in %BOP, mean PD and mean CAL between treatment and control group at 3 m
Mizuno [50] Japan	37 (28,9) NR 7	DM	NSPT; 20/5 61.2 ± 9.2 HI & US	OHI; 17/4 62.8 ± 12.1	Unknown Pr FM 6 spt	BL 3 m 6 m	Significant difference in mean PD and mean CAL between treatment and control group at 3 m and 6 m follow-up, for %BOP only at 6 m
Montenegro [51] Brazil	82 (21,61) NR 9	CVD	NSPT; 43/4 58.4 ± 9.2 NR	SGS; 43/0 60.8 ± 8.5	Williams Pr FM excl 3 M 6 spt	BL 3 m	Significant difference in %BOP, mean PD and mean CAL between treatment and control group at 3 m
Offenbacher [52] USA	74 (0,74) NR 7*	Pregnancy	NSPT; 40/15 26.8 ± 5.5* NR	SGS; 34/6 25.7 ± 5.4*	UNC 15 Pr FM 6 spt	BL 3 m	Significant difference in %BOP, mean PD and mean CAL between treatment and control group at 3 m
Offenbacher ^†^ [53] USA	303 (NR) NR 51*	CVD	NSPT; 151/25 59.5* US	OHI; 152/50 59.8*	NR FM NR	BL 6 m 12 m	Significant difference in mean PD and mean CAL between control and treatment group at 3 m; no significant difference in %BOP
Pham [54] Vietnam	42 (42,0) 54.3 ± 7.4 NR	DM	NSPT + CHX; 21/1 53.5 ± 8.0 HI & US	SGS + CHX; 21/1 55.1 ± 6.8	UNC Pr NR 6 spt	BL 3 m 6 m	Significant difference in %BOP, mean PD and mean CAL between treatment and control group at 3 m and 6 m
Qureshi [55] Pakistan	100 (52,48) NR 6	DM	NSPT; 50/17 51.24 ± 18.27 HI & US	OHI; 50/18 52.82 ± 6.38	NR NR NR	BL 3 m	Significant difference in %BOP, mean PD and mean CAL between treatment and control group at 3 m
Raman [56] Malaysia	32 (20,12) NR Excluded	DM	NSPT; 20/5 57.7 ± 9:9 HI & US	OHI; 20/3 54.6 ± 6.2	Florida Pr NR NR	BL 2 m 3 m	No significant difference in mean PD and mean CAL between treatment and control group at 3 m
Saffi [57] Brazil	69 (52,17) NR 8	CVD	NSPT; 31/0 58.6 ± 8.5 NR	No Tx; 38/0 61.7 ± 8.3	Williams Pr FM excl 3 M 6 spt	BL 3 m	Significant difference in %BOP, mean PD and mean CAL between treatment and control group at 3 m
Wang S [58] China	39 (26,13) NR 9	DM	NSPT; 22/3 61.58 ± 4.6 NR	No Tx; 22/2 61.90 ± 6.7	Williams Pr FM 6 spt	BL 3 m	Significant difference in mean PD and mean CAL between treatment and control group at 6 m
Wang Y [59] China	18 (9,9) NR 2	DM	NSPT; 11/0 65 ± 8 HI & US	OHI; 7/1 68 ± 3	NR FM excl 3 M 6 spt	BL 6 m	Significant difference in %BOP between treatment and control group at 6 m
Wu [60] China	46 (22,24) NR Excluded	DM	NSPT; 27/4 54.09 ± 6.5 NR	OHI; 27/4 55.52 ± 5.2	Unknown Pr FM NR	BL 3 m 6 m	Significant difference in mean PD and mean CAL between treatment and control group at 3 m and 6 m

*M* males, *F* females, *CAL* clinical attachment level (mm), *PD* probing pocket depth (mm), *SD* standard deviation, *NSPT* non-surgical periodontal treatment, *SGS* supragingival scaling, *OHI* oral hygiene instructions, *CHX* chlorhexidine mouthwash, *Tx* treatment, *DM* diabetes mellitus, *CVD* cardiovascular disease, *PCOS* polycystic ovarian syndrome, *ED* erectile dysfunction, *COPD* chronic obstructive pulmonary disease, *CKD* chronic kidney disease, *RA* rheumatoid arthritis, *CRP* C-reactive protein, *Chol* cholesterol, *FM* full mouth, *3 M* third molars, *excl* excluding, *m* month, *NR* not reported, *HI* hand instruments, *US* ultrasonic scaling, *Pr* probe, *UNC* University of North Carolina, *NC* North Carolina, *spt* sites per tooth

^*^Data from baseline intention-to-treat patient population; ^#^Data from baseline per-protocol patient population

^†^The study was included only in the risk of bias assessment and study characteristics table, but excluded from the meta-analysis

Twenty-seven studies involving 2,530 participants and thirteen studies involving 1,292 patients reported mean CAL at the 3-month and 6-month examinations, respectively. Thirty studies involving 2,826 patients and sixteen studies involving 1,470 patients reported mean PD at the 3-month and 6-month examinations, respectively. Twenty-three studies involving 2,333 patients and fifteen studies involving 1,424 patients reported %BOP at the 3-month and 6-month examinations, respectively.

### Risk of bias

**Fig. 1:**
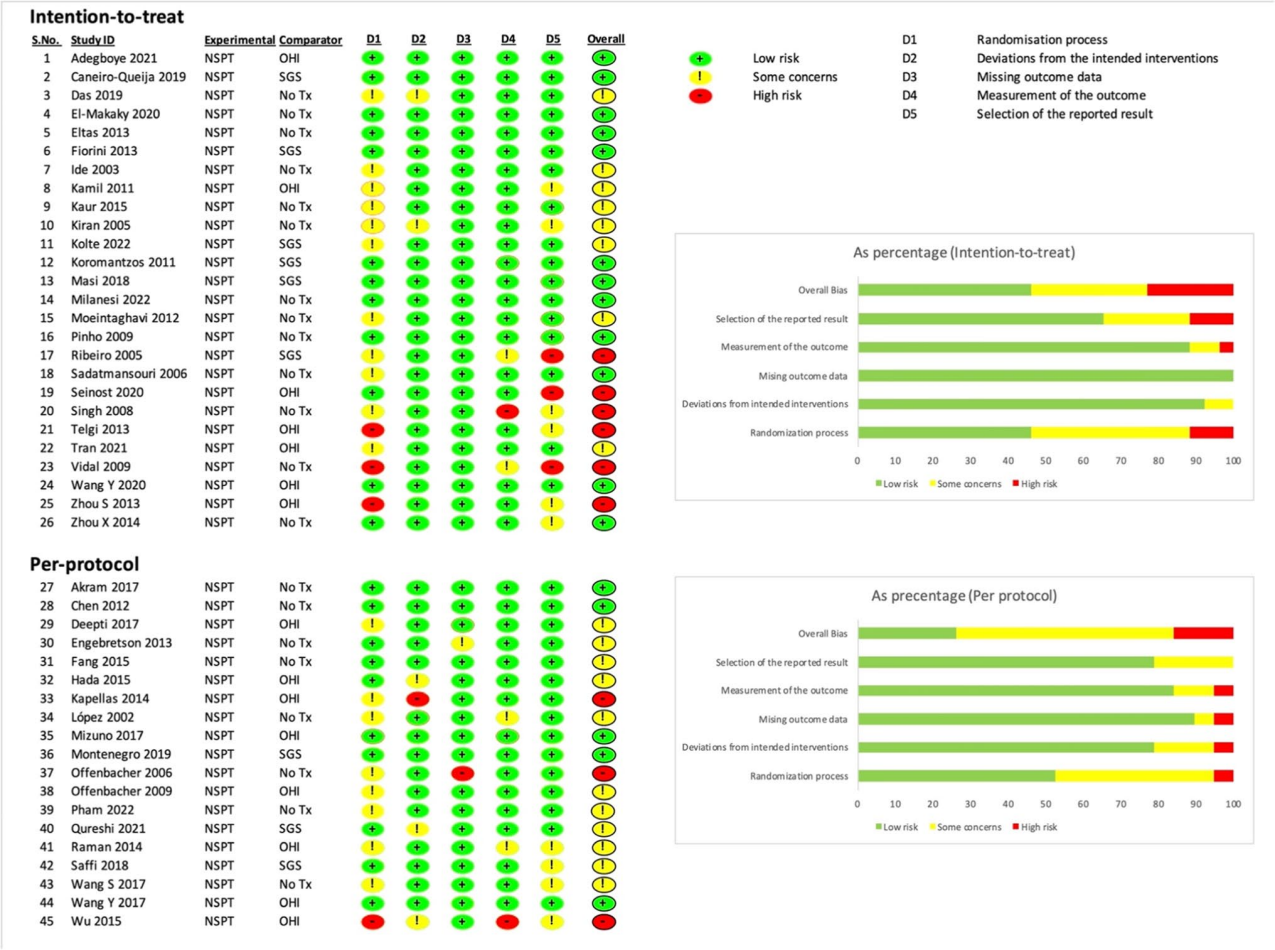
Risk of bias assessment for all included studies and summarized based on the intention-to-treat and per-protocol criteria

The risk of bias assessment was summarized based on the intention-to-treat or per-protocol principle (Fig. 1). The risk of bias for all domains was low in 17 trials. In the remaining 19 trials, the risk of bias was of some concern because it was not explicitly described whether sequence generation and/or allocation concealment was adequately done. Nine studies had a high risk of bias, because protection against performance and detection biases was inadequate, as personnel and outcome assessment were unblinded or not mentioned. Blinding patients to the intervention was impossible due to the nature of the interventions. Evaluations of a potential publication bias revealed a significant small-study effect for PD reduction. Twenty-six studies analysed all patients, 19 studies only post-treatment data from patients who were available at a follow-up visit, and 21 studies reported an analysis based on intention-to-treat. Compliance with treatment was not a concern given that most studies performed SRP once at baseline. Because the selected studies were not planned to evaluate the effect of NSPT versus no NSPT, we did not include this aspect in our bias assessment.

### Meta-analysis

#### Probing depth

**Fig. 2:**
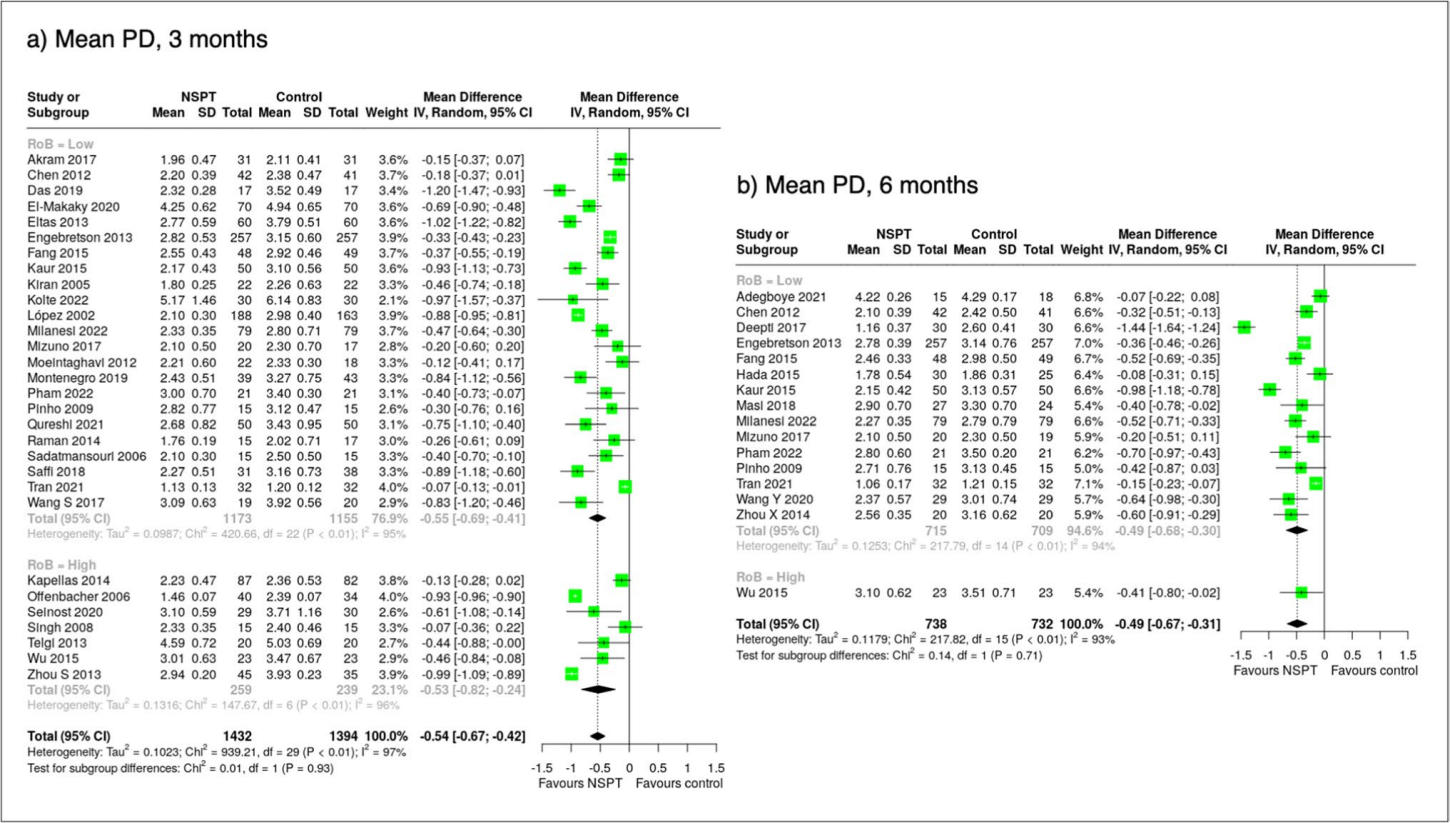
Forest plot showing the mean differences of mean probing depth (in mm) sorted according to risk of bias assessment (low/some concerns vs. high) at 3 and 6 months

In total, 2,826 patients from 30 studies with 3 months data with a high risk of bias, some concerns or low risk were analysed. Restricting 23 studies to those with low or some bias concerns showed a significant mean difference in mean PD of −0.55 mm (95% C.I.: −0.69; −0.41) favouring NSPT (Fig. 2). Including all studies, irrespective of bias, did not change this mean difference. A subgroup analysis with diabetic patients yielded similar results, with a mean PD difference of −0.49 mm (95% C.I.: −0.68; −0.31) in favour of NSPT. Restricting studies to those with CVD patients and low or some concerns of bias (2 studies with 151 CVD patients) yielded a mean PD difference of −0.86 mm (95% C.I.: −1.06; −0.66) in favour of NSPT (Table 2).

**Table 2:** Pooled mean differences of outcome variables stratified by the systemic disease/ condition, and further stratified based on the risk of bias score

Systemic disease/condition		At 3 months				At 6 months		
	Risk of bias	Mean PD, mm	Mean CAL, mm	%BOP, %	%PD ≤ 3 mm, %*	Mean PD, mm	Mean CAL, mm	%BOP, %
Cardiovascular diseases	Low or some concerns	−0.86 (−1.06; −0.66)	−0.56 (−0.97; −0.15)	−35.00 (−47.47; −22.53)	24.01 (3.31; 44.72)		−0.46 (−0.77; −0.14)	
	High	−0.58 (−1.10; −0.05)	−0.55 (−0.75; −0.36)	−18.70 (−25.58; −11.81)	9.55 (−4.22; 23.32)		−0.76 (−1.43; −0.09)	
	Overall	−0.69 (−1.02; −0.36)	−0.56 (−0.73; −0.38)	−28.18 (−38.48; −17.88)	16.89 (3.58; 30.19)		−0.49 (−0.77; −0.21)	
Diabetes mellitus	Low or some concerns	−0.49 (−0.68; −0.31)	−0.54 (−0.72; −0.36)	−18.70 (−26.87; −10.53)	13.76 (3.79; 23.73)	−0.47 (−0.65; −0.29)	−0.49 (−0.68; −0.30)	−26.44 (−33.75; −19.13)
	High	−0.29 (−0.57; −0.02)	−0.19 (−1.25; 0.88)	–	–	−0.41 (−0.80; −0.02)	−0.76 (−1.43; −0.09)	–
	Overall	−0.46 (−0.63; −0.30)	−0.48 (−0.69; −0.28)	−18.70 (−26.87; −10.53)	13.76 (3.79; 23.73)	−0.46 (−0.63; −0.30)	−0.51 (−0.69; −0.32)	−26.44 (−33.75; −19.13)
Erectile dysfunction	Low or some concerns	−0.86 (−1.18; −0.53)	−0.69 (−0.85; −0.52)	−38.31 (−45.90; −30.73)				
	High	–	–	–				
	Overall	−0.86 (−1.18; −0.53)	−0.69 (−0.85; −0.52)	−38.31 (−45.90; −30.73)				
Pregnancy	Low or some concerns	−0.66 (−1.13; −0.19)	−0.35 (−0.86; 0.16)		15.82 (5.11; 26.53)			
	High	−0.93 (−0.96; −0.90)	−0.13 (−0.15; −0.11)		–			
	Overall	−0.77 (−1.06, −0.47)	−0.29 (−0.67; 0.08)		15.82 (5.11; 26.53)			
Rheumatoid arthritis	Low or some concerns			−8.34 (−25.73; 9.05)				
	High			−28.90 (−42.12; −15.68)				
	Overall			−19.43 (−39.51; 0.66)				

^*^ This analysis was performed by including the studies where standard deviations were imputed

At 6 months, using data from 15 trials (1,424 patients) with low or some concerns of bias, a mean PD difference of −0.49 mm (95% C.I.: −0.68; −0.30) was observed between the NSPT and the control group (Fig. 2). In diabetic patients (8 trials with low bias or some concerns and 1155 patients) a mean PD difference of −0.47 mm (95% C.I.: −0.65; −0.29) was observed (Table 2). We judged the overall level of certainty in the evidence to be moderate based on the evidence profile. Irrespective of performed analyses, heterogeneity varied between 97% at 3 months and 93% at 6 months including studies with high bias.

#### Clinical attachment level

**Fig. 3:**
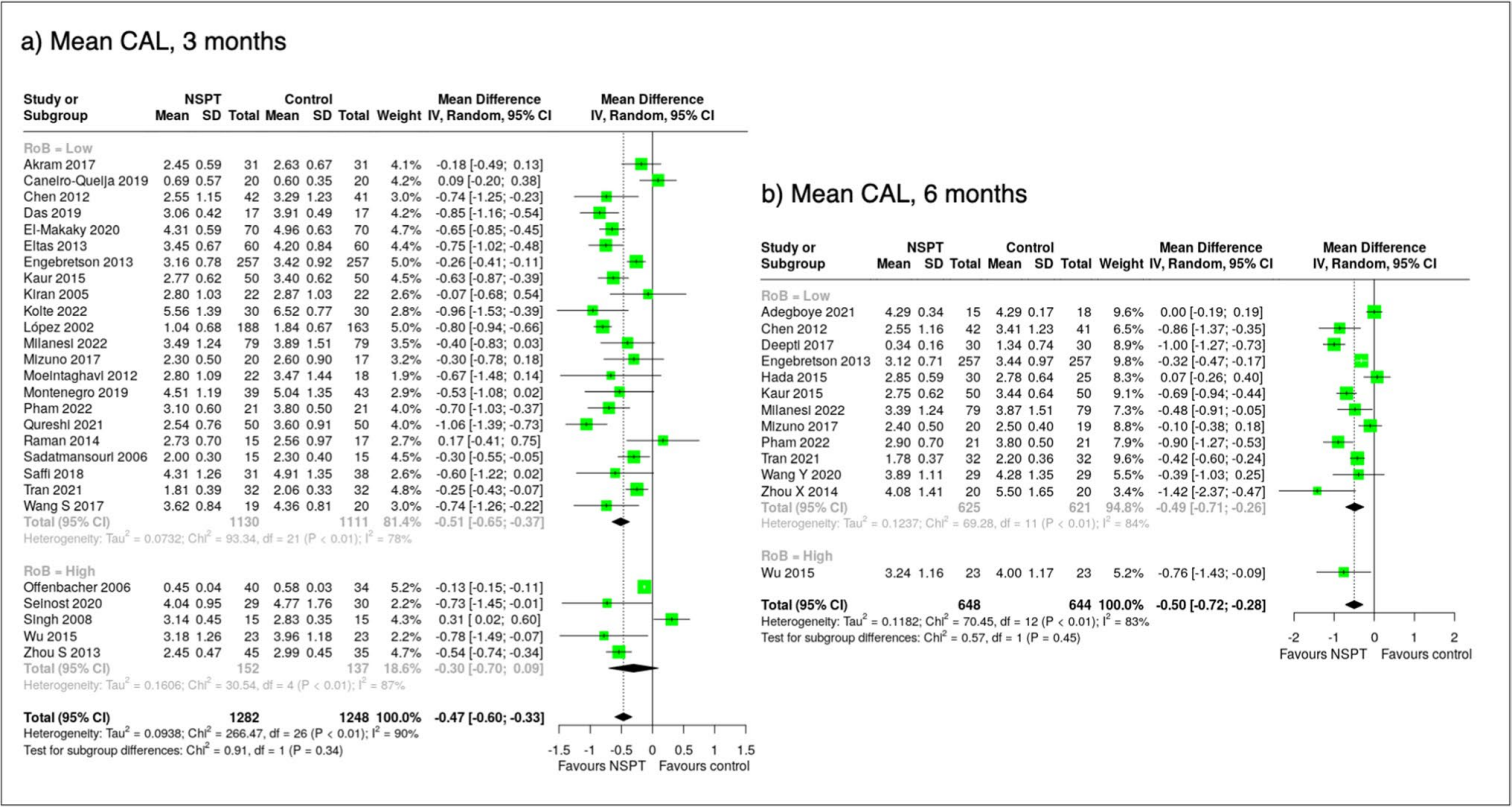
Forest plot showing the mean differences of mean clinical attachment loss (in mm) sorted according to risk of bias assessment (low/some concerns vs. high) at 3 and 6 months

In total, 2241 patients from 22 studies with 3-month data with low bias or some concerns were analysed. A statistically significant mean CAL difference of −0.51 mm (95% C.I.: −0.65; −0.37) in favour of NSPT was observed. Including five studies (289 patients) with high bias did not materially change the mean CAL difference (−0.30 mm (95% C.I.: −0.70; 0.09) (Fig. 3). Subgroup analysis yielded similar results: 14 studies with 1347 diabetic patients with low bias or some concern, the mean CAL difference was −0.54 mm (95% C.I.: −0.72; −0.36) in favour of NSPT. From two CVD (151 patients) studies with low bias or some concern, the mean CAL difference was −0.56 mm (95% C.I.: −0.97; −0.15) (Table 2).

At 6 months, 12 trials (1244 patients) with low bias or some concern showed a mean CAL difference of -0.49 mm (95% C.I.: −0.71; −0.26) in favour of NSPT (Fig. 3). Eight trials involving 1058 diabetic patients yielded a mean CAL difference of −0.49 mm (95% C.I.: −0.68; −0.30) in favour of NSPT (Table 2). Including all RCTs, the study heterogeneity was 90% at 3 months and 83% at 6 months.

#### Bleeding on probing

**Fig. 4:**
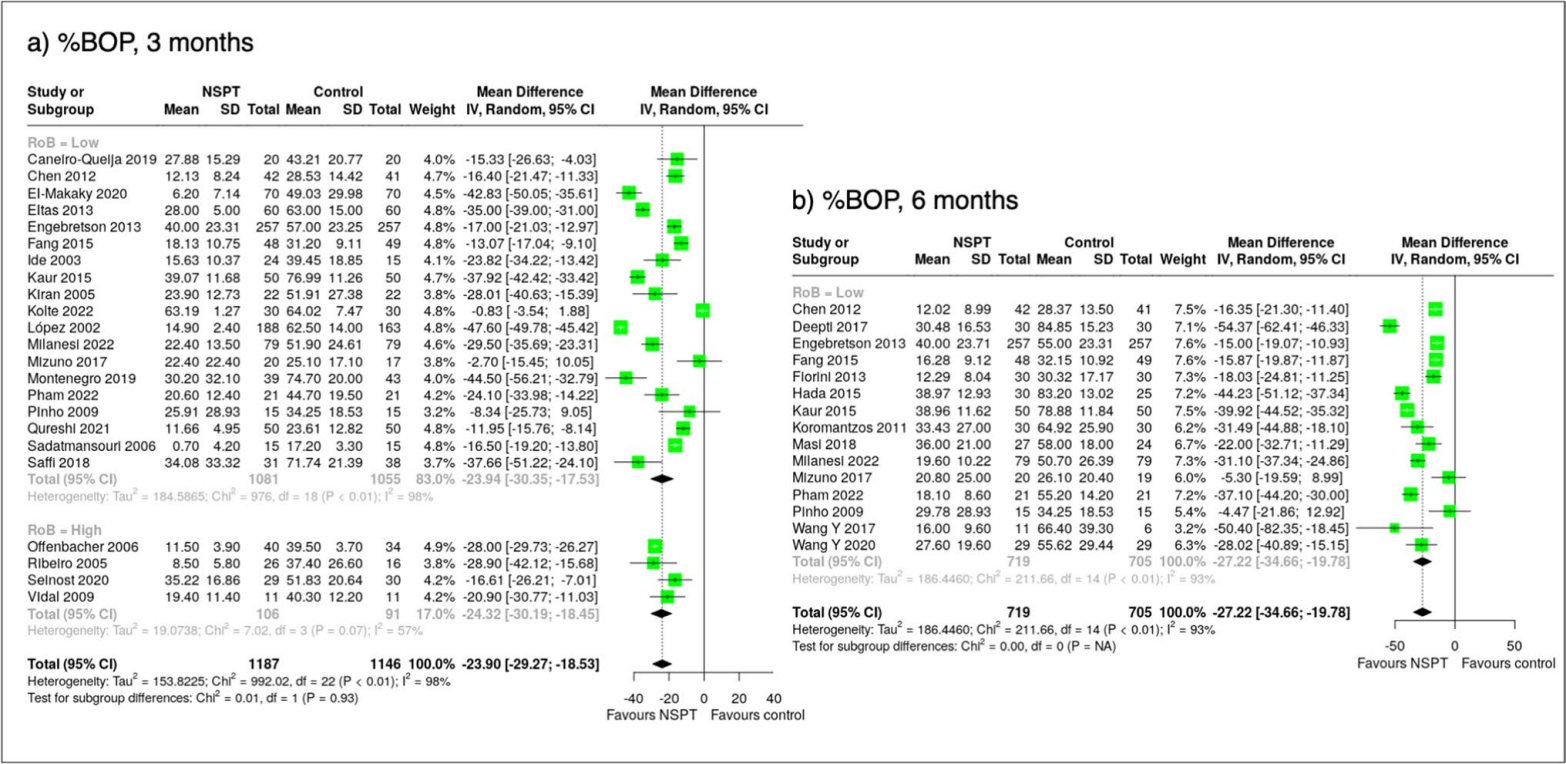
Forest plot showing the mean differences of the percentage of sites with bleeding on probing (%BOP) sorted according to risk of bias assessment (low/some concerns vs. high) at 3 and 6 months

In total, 19 studies (2,134 patients) with 3-month data with low bias or some concerns were analysed. %BOP was significantly lower (−23.94% (95% C.I.: −30.35%; −17.53%)) in NSPT compared to the control group. Including studies with high bias did not change the results (−23.90% (95% C.I.: −29.27; −18.53)) (Fig. 4). The subgroup analysis of nine diabetes studies (1138 patients) yielded similar results: the mean %BOP difference was −18.70% (95% C.I.: −26.87; −10.53), whereas in three CVD studies (190 patients), the mean %BOP difference was -35.00% (95% C.I.: −47.47; −22.53) (Table 2).

At 6 months, in 15 trials with low bias or some concerns, including 1,422 patients, a mean %BOP difference of −27.22% (95% C.I.: −34.66; −19.78) was observed in favour of NSPT (Fig. 4). 10 trials with 1120 diabetic patients yielded a mean %BOP difference of −26.44% (95% C.I.: −33.75; −19.13) in favour of NSPT (Table 2). Study heterogeneity was 98% at 3 months and 93% at 6 months, including all RCTs, showing considerable significance.

#### Percentage of sites with probing depth ≤ 3 mm

**Fig. 5:**
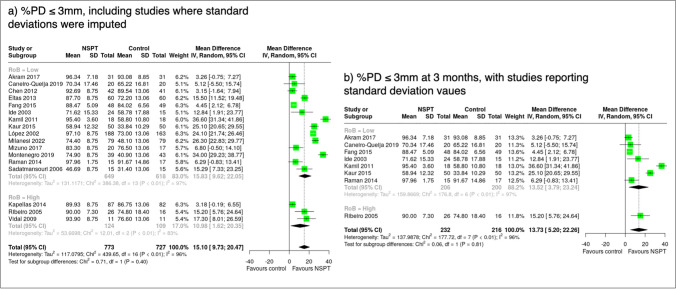
Forest plot showing the mean differences of the percentage of sites with probing depths ≤ 3 mm (%PD ≤ 3 mm) sorted according to risk of bias assessment (low/some concerns vs. high) at 3 months; **a** including the studies where missing standard deviations were imputed; **b** only studies with complete information

Because of the limited number of studies at 6-month follow-up with this information, the results in this section were limited to the 3-month follow-up. Eight studies (one with high risk of bias) provided means and standard deviations. The overall mean difference in %PD ≤ 3 mm between NSPT and the control group was 13.73% (95% C.I.: 5.20; 22.26). When unreported standard deviations were imputed, 16 studies (3 with high risk of bias) were included. The mean difference in %PD ≤ 3 mm was 14.98% (95% C.I.: 8.48; 21.48) in studies with low/some concern bias and 14.36% (95% C.I.: 8.83; 19.89) in all a. However, the pooled estimates were lower (10.98% (95% C.I.: 1.62; 20.35)) in high-risk studies (Fig. 5).

#### Pre-and post-treatment results

Irrespective of bias and treatment arm, mean PD at baseline varied between 1.21 ± 0.27 mm and 6.59 ± 1.50 mm (Appendix Table 3). NSPT reduced mean PD with a mean pre-post difference of 0.56 mm (95% C.I.: 0.46; 0.66) and 0.58 mm (95% C.I.: 0.40; 0.76) at 3 and 6 months, respectively (Appendix Fig. 2). Mean CAL reduced with a mean pre-post difference of 0.50 mm (95% C.I.: 0.38; 0.62) and 0.41 mm (95% C.I.: 0.21; 0.60) at 3 and 6 months, respectively. %BOP reduced with a mean pre-post difference of 29.92 (95% C.I.: 23.97; 35.87) and 32.28 (95% C.I.: 26.04; 38.52) at 3 and 6 months, respectively. In the NSPT group, %PD ≤ 3 mm increased by 17.32% (95% C.I.;23.80; 10.84) between baseline and the 6-month examination.

### Meta-regression

We performed random-effects meta-regression analyses including all studies with 3- or 6-month follow-up data (Appendix Table 4 and 5, respectively). None of the factors were significantly associated with the meanPD at 3-month follow-up; but only a comorbidity type (PCOS: β = −0.743 (95% C.I.: −1.411; −0.076)) was found to be significantly associated with mean PD at 6-month follow-up (Appendix Table 4 and Appendix Table 5).

## Discussion

This systematic review aimed to summarise the current literature on the efficacy of NSPT compared to no or minimal periodontal treatment in patients with comorbidities. Consistent with previous reviews on this topic, our primary outcome was mean PD, while our secondary outcomes included mean CAL, percentage BOP and percentage of sites with PD ≤ 3 mm assessed at 3 and 6 months. We acknowledge that mean PD may not have clear clinical relevance, but most included studies reported mean PD across all sites. In contrast, only very few reported the percentage of sites with PD 4–5 mm or ≥ 6 mm, which more accurately depicts the clinical situation. NSPT showed a 0.55 mm (95% C.I.: −0.69, −0.41) lower mean PD at the 3-month examination than the control group when studies were restricted to those with low bias or some concerns. The difference in mean PD attenuated to −0.49 mm (95% C.I.: −0.68, −0.30) at the 6-month examination.

Even when studies with a high risk of bias were included in the analysis, there was no change in mean PD difference. Regardless of the underlying comorbidity (diabetes, CVD, pregnancy), mean PD differences were consistent. Analyses of secondary outcomes (mean CAL, %BOP and %PD ≤ 3 mm) support our conclusion that NSPT reduced periodontal inflammation in periodontitis patients with comorbidities. The significant variation of baseline mean PD levels reflect the different inclusion criteria for periodontitis (Appendix Table 3). Although the RCTs included in our meta-analysis were primarily not designed to answer the above formulated question, namely the effect of NSPT in comparison to no treatment, SGS or OHI on periodontal conditions, we feel confident that our conclusion is robust. From our perspective, these results provide a definitive ‘yes’ that NSPT is an effective therapeutic measure in terms of clinical outcomes.

Overall, few previous reviews have investigated the question of whether NSPT is superior to no treatment, SGS or OHI, but results did not provide definitive conclusions due to the ethical implications of withholding periodontal therapy in the control group. A first review that tried to shed light on this question found two studies in which the treatment arm gained 0.22 mm more mean CAL compared to untreated controls [2, 61, 62]. In addition, this review included an observational study with two arms: 79 periodontally diseased subjects with no periodontal treatment and 108 patients treated with NSPT were monitored for one year. Compared to baseline, mean PD reduced by 0.50 ± 0.04 mm and mean CAL decreased by 0.44 ± 0.05 mm in the treatment group; in the untreated group mean PD decreased by 0.04 ± 0.05 mm, while mean CAL decreased by 0.21 ± 0.21 mm [63]. These data are in line with our results.

An open question remains as to whether the statistically significant difference in mean PD and mean CAL of 0.5 mm between the test and control group at the final examination or the pre-post mean differences of about 0.57 and 0.53 mm for mean PD and mean CAL in the test group, respectively, are clinically relevant. Extent values give a better understanding of the clinical reality: the pre-post %PD ≤ 3 mm in the test group increased by about 18.04%, from 66.21% to 83.63%, whereas it was materially zero in the control group. In the treatment arm pre-post %BOP was reduced from 53.2% to 23.1%, whereas in the control arm, it only decreased from 51.7% to 46.9%. Although the patients included in this meta-analysis were less severely diseased than those in a multicentre RCT with 200 patients (Harks et al. 2015), the resolution of inflammation exhibited comparable healing trends. The NSPT arm of this multicentre RCT showed a decrease in mean PD from 3.5 ± 0.8 mm to 2.7 ± 0.7 mm, %BOP was reduced from 34.2 ± 18.1% to 19.6 ± 14.9%, and %PD ≤ 3 mm increased from 59.2 ± 18.1% to 79.1 ± 15.9% (Harks et al. 2015). These data align very well with the data reported here. From our perspective, these values reflect clinically notable results, but they are still far from meeting the criteria for a successfully treated periodontitis patient as defined by the 2017 Workshop [64], should only exhibit BOP in < 10% of sites and have no sites with PD ≥ 4 mm and bleeding on probing. These studies suggest that even under institutional conditions, it may be difficult to achieve such a threshold.

In 2019, the European Federation of Periodontology commissioned a meta-analysis on the efficacy of NSPT [6]. The authors restricted their inclusion criteria to patients without comorbidities and found only one study, which did not allow for any robust conclusions to be drawn. To still answer this basic question, they analysed studies with different treatment protocols and reported the pre-post-treatment change in mean PD. Their reported results are based on a mixture of all measured sites with only moderate pockets, which prevents comparing their results with ours.

To answer whether NSPT outcomes achieved in medically compromised patients are inferior to those achieved in systemically healthy patients, we compared our results with data extracted from 53 reports with 1,474 systemically healthy periodontitis patients who underwent NSPT [65]. Three months after NSPT, the initial mean PD of 3.9 mm was reduced by 0.78 mm (95% C.I: 0.76–0.79) and the mean CAL gain was 0.65 mm (95% C.I: 0.63–0.67).

**Fig. 6:**
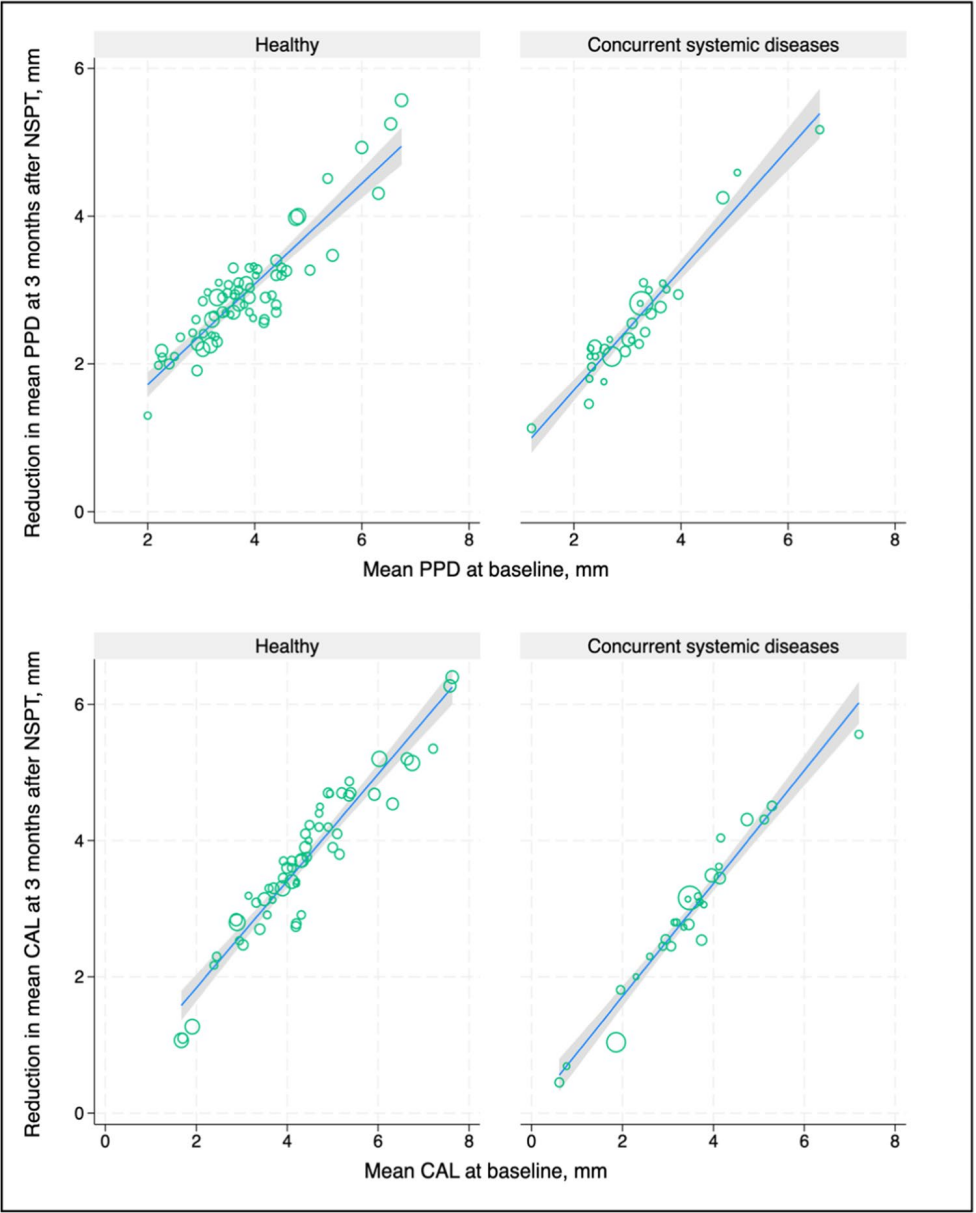
Irrespective of the comorbidity status, baseline values of mean PD and mean CAL were strongly associated with the corresponding measures at 3-months after NSPT. Study groups with higher mean PD at baseline exhibited a higher mean PD after therapy, whereas higher values of mean PD at baseline were concomitantly associated with greater reductions in mean PD over 3 months. Regarding the shape of the association, no significant differences between systemically healthy and diabetes groups were observed. In analyses of mean CAL, analogous results were obtained. The circle sizes represent the respective study sizes. For detailed information about the studies included in the arm with healthy subjects, please refer to Kocher et al. 2018. [66]

To graphically support the comparability of the short-term results in treating patients with and without comorbidity, baseline values of mean PD and CAL were associated with the corresponding values 3 months after NSPT. The slope of the association of mean PD or mean CAL did not differ between systemically healthy individuals and those with a comorbidity (Fig. 6). Although this comparison does not allow for any statistical inference, our results suggest that NSPT in medically compromised patients may produce similar results as in systemically healthy patients.

One strength of this review, which contributes to the robustness of the conclusion, is the high external validity base, as the patients participating in the RCTs were representative of the general population because they were not recruited in a dental school but rather from hospitals with different specialties.

This meta-analysis has several limitations. First, these studies were performed in periodontal institutions with presumably high technical scaling skills. Therefore, the present review describes the efficacy of the intervention rather than its effectiveness and does not reflect periodontal treatment in the community. Second, this meta-analysis provides robust estimates for NSPT efficacy only for a 3- and 6-month period, which is definitely too short to determine if NSPT has a long-term effect. One single-centre study and one large multicentre study reported stable mean PD reduction or mean CAL gain after NSPT without antibiotics after 12 or 27.5 months [67, 68]. On the other hand, treatment effects after 3 months are not blurred by periodontal maintenance measures. Third, with the delayed treatment design, the control arm was often offered supragingival cleaning and OHIs to motivate the patients to stay in the RCT instead of no treatment. But even this very first treatment step of OHI might cause a considerable resolution of periodontitis [69]. Thus, only considering the difference between periodontal variables between the test and control arm at the final visit may be misleading because it neglects the influence of improved supragingival plaque control either to the professional motivation and instruction and/or the removal of supragingival calculus. Fourth, included studies were designed for research questions other than the one we set for this review. According to the bias assessment, most studies had some concerns or a high risk of bias. However, the D1 and D4 domains were of utmost importance for this review, and both these domains had little bias. Fifth, only limited information about the number of sessions or time spent on NSPT or OHI was provided. Sixth, information about drug intake was sparse and too diverse to consider its impact on treatment outcomes. However, because all the studies included in this review are RCTs, the impact of medications should be the same in the control and treatment arms. Seventh, information on smoking was not available in 21 studies. Thus, we could not address the confounding effects of smoking on treatment outcomes.

## Conclusion

There was a clinically relevant decrease in mean PD, mean CAL, and %BOP while having an increase in %PD ≤ 3 mm. Therefore, despite some limitations, this review’s findings suggest that NSPT is an effective procedure for managing periodontitis in patients with systemic diseases, which might be comparable with systemically healthy patients.

### Supplementary Information

Below is the link to the electronic supplementary material.

Supplementary file1 (DOCX 1217 KB)

## Data Availability

No new data was generated for this review. All the data used for the meta-analysis could be obtained directly from the respective publications. As a result, data sharing does not apply in this case.
