# Supplementary material for: Systematic review and meta-analysis of randomized controlled trials evaluating the efficacy of non-surgical periodontal treatment in patients with concurrent systemic conditions
Source: Clin Oral Investig. 2023 Dec 26;28(1):21. doi: 10.1007/s00784-023-05392-6 (PMC10751251; doi:10.1007/s00784-023-05392-6)

**Systematic Review and Meta-Analysis of Randomized Controlled Trials on the Efficacy of Non-Surgical Periodontal Treatment in Patients with Concurrent Systemic Conditions**

Prabhakar Joseph, Priya Prabhakar, Birte Holtfreter, Jeanie Suvan, Thomas Kocher, Vinay Pitchika

**Supplementary material**

**Appendix Table 1.** Search algorithms for Pubmed and Embase.

| Pubmed | (((("Periodontitis"[Mesh] OR Periodontitides[tiab] OR Pericementitis[tiab] OR "gum disease*"[tiab] OR "periodontal disease*"[tiab] OR/AND (periodont* AND (syst* disease OR healthy OR dysfunction OR diabet* OR type 2 diabet* or type II diabet* OR "Hyperlipidemias"[Mesh] OR "Vitamin D Deficiency"[Mesh] OR "Obesity"[Mesh] OR "Cardiovascular Diseases"[Mesh] OR "Respiratory Tract Diseases"[Mesh] OR "Arthritis, Rheumatoid"[Mesh] OR "bone diseases, metabolic"[MeSH Terms] OR "Genetic Predisposition to Disease"[Mesh] OR tumour OR cancer OR "HIV Infections"[Mesh] OR "Pregnancy"[Mesh] OR "Menopause"[Mesh]))) AND ("Biological Factors"[Mesh] OR "Biologic Marker*"[tiab] OR "Biological Marker*"[tiab] OR "Laboratory Marker*"[tiab] OR "Serum Marker*"[tiab] OR "Surrogate Endpoint*"[tiab] OR "Surrogate End Point*"[tiab] OR "Clinical Marker*"[tiab] OR glycaemic OR glycemic OR metabolic OR "Viral Marker*"[tiab] OR "Biochemical Marker*"[tiab] OR "Immune Marker*"[tiab] OR "Immunologic Marker*"[tiab] OR "Surrogate Marker*"[tiab] OR "outcome")) AND ("Periodontal Debridement"[Mesh] OR "Dental Scaling*"[tiab] OR "Root Scaling*"[tiab] OR "Subgingival Scaling*"[tiab] OR "Periodontal Debridement*"[tiab] OR "Nonsurgical Periodontal Debridement*"[tiab] OR "Periodontal Pocket Debridement*"[tiab] OR "Root Planing*"[tiab] OR "Subgingival Curettage*"[tiab] OR "Gingival Curettage*"[tiab] OR "scaling and root planning"[tiab] OR "periodontal therapy"[tiab] OR "periodontal treatment"[tiab] OR “PD treatment”)) NOT ("implant" OR "review")) |
| --- | --- |
| Embase | "Periodontitis" OR Periodontitides OR Pericementitis OR "gum disease*" OR "periodontal disease*" OR/AND periodont* AND syst* disease OR healthy OR dysfunction OR diabet* OR type 2 diabet* or type II diabet* OR "Hyperlipidemias" OR "Vitamin D Deficiency" OR "Obesity" OR "Cardiovascular Diseases" OR "Respiratory Tract Diseases" OR "Arthritis, Rheumatoid" OR "bone diseases, metabolic" OR "Genetic Predisposition to Disease" OR tumour OR cancer OR "HIV Infections" OR "Pregnancy" OR "Menopause" AND "Biological Factors" OR "Biologic Marker*" OR "Biological Marker*" OR "Laboratory Marker*" OR "Serum Marker*" OR "Surrogate Endpoint*" OR "Surrogate End Point*" OR "Clinical Marker*" OR glycaemic OR glycemic OR metabolic OR "Viral Marker*" OR "Biochemical Marker*" OR "Immune Marker*" OR "Immunologic Marker*" OR "Surrogate Marker*" OR "outcome" AND "Periodontal Debridement" OR "Dental Scaling*" OR "Root Scaling*" OR "Subgingival Scaling*" OR "Periodontal Debridement*" OR "Nonsurgical Periodontal Debridement*" OR "Periodontal Pocket Debridement*" OR "Root Planing*" OR "Subgingival Curettage*" OR "Gingival Curettage*" OR "scaling and root planning" OR "periodontal therapy" OR "periodontal treatment" OR “PD treatment” NOT "implant" OR "review" |

**Appendix Table 2:** Studies excluded after full-text reading, and the reasons for their exclusion from the meta-analysis.

| **Study** | **Reason for exclusion** |
| --- | --- |
| 1. Herrera, Julián A. et al. Periodontal intervention effects on pregnancy outcomes in women with preeclampsia. Colomb. Med. [online]. 2009, vol.40, n.2 [cited 2022-12-27], pp.177-184. 2. Jeffcoat, M. K., et al. (2003). Periodontal disease and preterm birth: results of a pilot intervention study. J Periodontol 74(8): 1214-1218. 3. Jeffcoat, M., et al. (2011). Periodontal infection and preterm birth: successful periodontal therapy reduces the risk of preterm birth. Bjog 118(2): 250-256. 4. Jones, J. A., et al. (2007). Does periodontal care improve glycemic control? The Department of Veterans Affairs Dental Diabetes Study. J Clin Periodontol 34(1): 46-52. 5. Macones GA, Parry S, Nelson DB, Strauss JF, Ludmir J, Cohen AW, Stamilio DM, Appleby D, Clothier B, Sammel MD, Jeffcoat M. Treatment of localized periodontal disease in pregnancy does not reduce the occurrence of preterm birth: results from the Periodontal Infections and Prematurity Stud y (PIPS). Am J Obstet Gynecol. 2010 Feb;202(2): 147.e1-8. 6. Michalowicz, B. S., et al. (2006). Treatment of periodontal disease and the risk of preterm birth. N Engl J Med 355(18): 1885-1894. 7. Michalowicz, B. S., et al. (2008). Examining the safety of dental treatment in pregnant women. J Am Dent Assoc 139(6): 685-695. 8. Michalowicz, B. S., et al. (2009). Serum inflammatory mediators in pregnancy: changes after periodontal treatment and association with pregnancy outcomes. J Periodontol 80(11): 1731-1741. 9. Moreira, C. H., et al. (2015). Periodontal treatment outcomes during pregnancy and postpartum. Clin Oral Investig 19(7): 1635-1641. 10. Musskopf, M. L., et al. (2018). Oral health related quality of life among pregnant women: a randomized controlled trial. Braz Oral Res 32: e002. 11. Newnham, J. P., et al. (2009). Treatment of periodontal disease during pregnancy: A randomized controlled trial. Obstet Gynecol 114(6): 1239-1248. 12. Novak, M. J., et al. (2008). Periodontal bacterial profiles in pregnant women: response to treatment and associations with birth outcomes in the obstetrics and periodontal therapy (OPT) study. J Periodontol 79(10): 1870-1879. 13. Oliveira, A. M., et al. (2011). Periodontal therapy and risk for adverse pregnancy outcomes. Clin Oral Investig 15(5): 609-615. 14. Penova-Veselinovic, B., et al. (2015). Changes in inflammatory mediators in gingival crevicular fluid following periodontal disease treatment in pregnancy: relationship to adverse pregnancy outcome. J Reprod Immunol 112: 1-10. 15. Radnai, M., et al. (2009). Benefits of periodontal therapy when preterm birth threatens. J Dent Res 88(3): 280-284 16. Tarannum, F. and M. Faizuddin (2007). Effect of periodontal therapy on pregnancy outcome in women affected by periodontitis. J Periodontol 78(11): 2095-2103 17. Weidlich, P., et al. (2013). Effect of nonsurgical periodontal therapy and strict plaque control on preterm/low birth weight: a randomized controlled clinical trial. Clin Oral Investig 17(1): 37-44. 18. Penova-Veselinovic B, Keelan JA, Wang CA, Newnham JP, Pennell CE. Changes in inflammatory mediators in gingival crevicular fluid following periodontal disease treatment in pregnancy: relationship to adverse pregnancy outcome. J Reprod Immunol. 2015 Nov;112:1-10. doi: 10.1016/j.jri.2015.05.002. Epub 2015 May 27. PMID: 26093363. 19. Pirie M, Linden G, Irwin C. Intrapregnancy non-surgical periodontal treatment and pregnancy outcome: a randomized controlled trial. J Periodontol. 2013 Oct;84(10):1391-400. doi: 10.1902/jop.2012.120572. Epub 2012 Dec 13. PMID: 23237583. 20. Merchant AT, Liu J, Reynolds MA, Beck JD, Zhang J. Quantile regression to estimate the survivor average causal effect of periodontal treatment effects on birthweight and gestational age. J Periodontol. 2021 Jul;92(7):975-982. doi: 10.1002/JPER.20-0376 Add to Citavi project by DOI. Epub 2020 Nov 5. PMID: 33155296. 21. Bose C, Valentine GC, Philips K, Boggess K, Moss K, Barros SP, Marchesan J, Wu D, O'Shea TM, Peralta-Carcelen M, Goldstein R, Ramamurthy R, Beck JD. Antepartum periodontitis treatment and risk of offspring screening positive for autism spectrum disorder. J Perinatol. 2023 Apr;43(4):470-476. doi: 10.1038/s41372-023-01610-x Add to Citavi project by DOI. Epub 2023 Jan 25. PMID: 36697694. | Non-criteria follow-up duration (n=21) |
| 1. Abou-Raya, A., et al. (2010). Periodontal disease, systemic inflammation and adipocytokines: Effect of periodontal therapy on glycaemic control in type 2 diabetes mellitus (T2DM) patients. Diabetes, Obesity and Metabolism 12: 76. 2. Agarwal, E., et al. (2017). Locally delivered 0.5% azithromycin as an adjunct to non-surgical treatment in patients with chronic periodontitis with type 2 diabetes: a randomized controlled clinical trial. J Periodontol 88(12): 1281-1287. 3. Ardila, C. M., et al. (2015). Adjunctive moxifloxacin in the treatment of generalized aggressive periodontitis patients: clinical and microbiological results of a randomized, triple-blind and placebo-controlled clinical trial. J Clin Periodontol 42(2): 160-168 4. Bajaj, P., et al. (2012). Locally delivered 0.5% clarithromycin, as an adjunct to nonsurgical treatment in chronic periodontitis with well-controlled type 2 diabetes: a randomized controlled clinical trial. J Investig Clin Dent 3(4): 276-283. 5. Basegmez, C., et al. (2011). Clinical and biochemical efficacy of minocycline in nonsurgical periodontal therapy: A randomized controlled pilot study. Journal of Clinical Pharmacology 51(6): 915-922. 6. Bhatia, A., et al. (2015). A randomized clinical trial of salivary substitute as an adjunct to scaling and root planing for management of periodontal inflammation in mouth breathing patients. J Oral Sci 57(3): 241-247. 7. Bizzarro, S., et al. (2017). Effect of periodontal therapy with systemic antimicrobials on parameters of metabolic syndrome: A randomized clinical trial. J Clin Periodontol 44(8): 833-841. 8. Bregy, L., et al. (2019). Metabolic changes during periodontitis therapy assessed by real-time ambient mass spectrometry. Clinical Mass Spectrometry 14: 54-62. 9. Campus, G., et al. (2007). Clinical effects of mechanical periodontal therapy in type 2 diabetic patients. Diabetes Res Clin Pract 75(3): 368-369. 10. Castro dos Santos, N. C., et al. (2020). Omega-3 PUFA and aspirin as adjuncts to periodontal debridement in patients with periodontitis and type 2 diabetes mellitus: Randomized clinical trial. J Periodontol 91(10): 1318-1327. 11. Christodoulides, N., et al. (2008). Photodynamic therapy as an adjunct to non-surgical periodontal treatment: A randomized, controlled clinical trial. J Periodontol 79(9): 1638-1644. 12. Dhingra, M. S., et al. (2010). Effect of periodontal therapy on serum c -reactive protein levels. Biomedicine 30(3): 286-291. 13. Fenol, A., et al. (2017). Correlation of salivary neopterin and plasma fibrinogen levels in patients with chronic periodontitis and/or type 2 diabetes mellitus. Pteridines 28(3-4): 177-183. 14. Gupta, S., et al. (2015). Effect of Time Lapse between Endodontic and Periodontal Therapies on the Healing of Concurrent Endodontic-Periodontal Lesions without Communication: A Prospective Randomized Clinical Trial. Journal of endodontics 41(6): 785-790. 15. Hasan, F., et al. (2019). Effectiveness of Simvastatin 1% oral gel and mouthwash used as an adjunct treatment of scaling and root planning in the treatment of periodontal diseases. Pak J Pharm Sci 32(6): 2673-2677. 16. Preus, H. R., et al. (2013). A randomized, double-masked clinical trial comparing four periodontitis treatment strategies: 1-year clinical results. J Periodontol 84(8): 1075-1086. 17. Preus, H. R., et al. (2015). Microbiologic Observations After Four Treatment Strategies Among Patients with Periodontitis Maintaining a High Standard of Oral Hygiene: Secondary Analysis of a Randomized Controlled Clinical Trial. J Periodontol 86(7): 856-865. | No control group (n=17) |
| 1. Calabrese, N., et al. (2011). Effects of periodontal therapy on glucose management in people with diabetes mellitus. Diabetes and Metabolism 37(5): 456-459. 2. Çetiner, D., et al. (2019). The role of visfatin levels in gingival crevicular fluid as a potential biomarker in the relationship between obesity and periodontal disease. J Appl Oral Sci 27: e20180365. 3. Chandni, R., et al. (2015). Effect of nonsurgical periodontal therapy on insulin resistance in patients with type 2 diabetes mellitus and chronic periodontitis. Diabetes 64: A692. 4. Cheah, C. W., et al. (2020). Salivary and serum cathelicidin LL-37 levels in subjects with rheumatoid arthritis and chronic periodontitis. International Journal of Rheumatic Diseases 23(10): 1344-1352. 5. D'Aiuto, F., et al. (2004). Periodontitis and atherogenesis: Causal association or simple coincidence? A pilot intervention study. J Clin Periodontol 31(5): 402-411. 6. Joseph, R., et al. (2016). Effect of nonsurgical periodontal therapy on glycosylated hemoglobin levels in prediabetic patients with chronic periodontitis. Diabetes 65: A382. 7. Joseph, R., et al. (2017). Nonsurgical periodontal-therapy improves glycosylated hemoglobin levels in pre-diabetic patients with chronic periodontitis. World Journal of Diabetes 8(5): 213-221. 8. Kanduluru A, Naganandini S. Effect of nonsurgical periodontal treatment on clinical response and glycemic control in type 2 diabetic patients with periodontitis: Controlled clinical trial. J Indian Assoc Public Health Dent 2014;12:261-7 9. Li, X., et al. (2013). Periodontal therapy decreases serum levels of adipocyte fatty acid-binding protein in systemically healthy subjects: a pilot clinical trial. J Periodontal Res 48(3): 308-314. 10. Mammen J, Vadakkekuttical RJ, George JM, Kaziyarakath JA, Radhakrishnan C. Effect of non-surgical periodontal therapy on insulin resistance in patients with type II diabetes mellitus and chronic periodontitis, as assessed by C-peptide and the Homeostasis Assessment Index. J Investig Clin Dent. 2017 Aug;8(3). 11. Mariotti, G., et al. (2013). Chronic periodontitis and cardiovascular disease: A controlled clinical trial. European Journal of Inflammation 11(2): 459-467. 12. Popova, C., et al. (2017). Correlations between healing parameters and PGE2 expression levels in non-surgical therapy of chronic periodontitis. Journal of IMAB - Annual Proceeding (Scientific Papers) 23(4): 1758-1764. 13. Sant'Ana, A. C., et al. (2011). Periodontal treatment during pregnancy decreases the rate of adverse pregnancy outcome: a controlled clinical trial. J Appl Oral Sci 19(2): 130-136. 14. Siribamrungwong, M. and K. Puangpanngam (2012). Treatment of periodontal diseases reduces chronic systemic inflammation in maintenance hemodialysis patients. Ren Fail 34(2): 171-175. 15. Stewart, J. E., et al. (2001). The effect of periodontal treatment on glycemic control in patients with type 2 diabetes mellitus. J Clin Periodontol 28(4): 306-310. | Not a randomized clinical trial (n=15) |
| 1. Bukleta, D., et al. (2018). Impact of combined non-surgical and surgical periodontal treatment in patients with type 2 diabetes mellitus-a preliminary report randomized clinical study. Biomedical Research (India) 29(3): 633-639. 2. D'Aiuto, F., et al. (2018). Systemic effects of periodontitis treatment in patients with type 2 diabetes: a 12 month, single-centre, investigator-masked, randomised trial. Lancet Diabetes Endocrinol 6(12): 954-965. 3. Oz, S. G., et al. (2007). Beneficial effects of periodontal treatment on metabolic control of hypercholesterolemia. South Med J 100(7): 686-691. 4. Taylor, B., et al. (2010). The effect of initial treatment of periodontitis on systemic markers of inflammation and cardiovascular risk: a randomized controlled trial. Eur J Oral Sci 118(4): 350-356. | Surgical intervention (n=4) |
| 1. Daneshvar, S., et al. (2010). An interventional study on HbA1c and serum, high-sensitivity CRP after local anti-infectious periodontal treatment in type 2 diabetic patients with periodontal disease. Hormone Research in Paediatrics 74: 177. 2. El-Makaky, Y. and H. K. Shalaby (2020). The effects of non-surgical periodontal therapy on glycemic control in diabetic patients: A randomized controlled trial. Oral Dis 26(4): 822-829. 3. El-Makaky, Y. and H. K. Shalaby (2020). The effects of non-surgical periodontal therapy on glycemic control in diabetic patients: A randomized controlled trial. Oral Dis 26(4): 822-829. 4. Fu, Y. W., et al. (2016). Effects of periodontal therapy on serum lipid profile and proinflammatory cytokines in patients with hyperlipidemia: a randomized controlled trial. Clin Oral Investig 20(6): 1263-1269. 5. Li, X., et al. (2011). Effect of periodontal treatment on circulating CD34(+) cells and peripheral vascular endothelial function: a randomized controlled trial. J Clin Periodontol 38(2): 148-156. 6. Tonetti, M. S., et al. (2007). Treatment of periodontitis and endothelial function. N Engl J Med 356(9): 911-920. 7. Tsobgny-Tsague, N. F., et al. (2018). Effects of nonsurgical periodontal treatment on glycated haemoglobin on type 2 diabetes patients (PARODIA 1 study): a randomized controlled trial in a sub-Saharan Africa population. | Use of antibiotics (n=7) |
| 1. Gao L, Sun XJ, Xie H, Nan SH, Xie HX. Effects of essential periodontal treatment on serum level of sCD40L and periodontal clinical parameters in patients with moderate to severe periodontitis at high risk of stroke. Shanghai Kou Qiang Yi Xue. 2016 Oct;25(5):574-578. Chinese. PMID: 28116430. 2. Radnai, M., et al. (2008). The possible effect of basic periodontal treatment on the outcome of pregnancy. Fogorv Sz 101(5): 179-185. 3. Zhong, Z. Q. and H. Fu (2018). [Effects of SPR periodontal basic therapy on cervical crevicular fluid C-reactive protein in patients with chronic periodontitis]. Shanghai Kou Qiang Yi Xue 27(5): 530-534. | Non-English language (n=3) |
| 1. Kapellas K, Do LG, Bartold PM, Skilton MR, Maple-Brown LJ, O'Dea K, Brown A, Celermajer DS, Slade GD, Jamieson LM. Effects of full-mouth scaling on the periodontal health of Indigenous Australians: a randomized controlled trial. J Clin Periodontol. 2013 Nov;40(11):1016-24. 2. Kapellas K, Mejia G, Bartold PM, Skilton MR, Maple-Brown LJ, Slade GD, O'Dea K, Brown A, Celermajer DS, Jamieson LM. Periodontal therapy and glycaemic control among individuals with type 2 diabetes: reflections from the PerioCardio study. Int J Dent Hyg. 2017 Nov;15(4):e42-e51. 3. Koromantzos PA, Makrilakis K, Dereka X, Offenbacher S, Katsilambros N, Vrotsos IA, Madianos PN. Effect of non-surgical periodontal therapy on C-reactive protein, oxidative stress, and matrix metalloproteinase (MMP)-9 and MMP-2 levels in patients with type 2 diabetes: a randomized controlled study. J Periodontol. 2012 Jan;83(1):3-10. doi: 10.1902/jop.2011.110148. 4. Mauri-Obradors, E., et al. (2018). Benefits of non-surgical periodontal treatment in patients with type 2 diabetes mellitus and chronic periodontitis: A randomized controlled trial. J Clin Periodontol 45(3): 345-353. 5. Reddy BV, Tanneeru S, Chava VK. The effect of phase-I periodontal therapy on pregnancy outcome in chronic periodontitis patients. J Obstet Gynaecol. 2014 Jan;34(1):29-32. 6. Artese HP, Longo PL, Gomes GH, Mayer MP, Romito GA. Supragingival biofilm control and systemic inflammation in patients with type 2 diabetes mellitus. Braz Oral Res. 2015;29:S1806-83242015000100266. doi: 10.1590/1807-3107BOR-2015.vol29.0071. 7. Caúla AL, Lira-Junior R, Tinoco EM, Fischer RG. The effect of periodontal therapy on cardiovascular risk markers: a 6-month randomized clinical trial. J Clin Periodontol. 2014 Sep;41(9):875-82. doi: 10.1111/jcpe.12290. Epub 2014 Aug 3. PMID: 25041550. 8. Offenbacher S, Beck JD, Moss K, Mendoza L, Paquette DW, Barrow DA, Couper DJ, Stewart DD, Falkner KL, Graham SP, Grossi S, Gunsolley JC, Madden T, Maupome G, Trevisan M, Van Dyke TE, Genco RJ. Results from the Periodontitis and Vascular Events (PAVE) Study: a pilot multicentered, randomized, controlled trial to study effects of periodontal therapy in a secondary prevention model of cardiovascular disease. J Periodontol. 2009 Feb;80(2):190-201. doi: 10.1902/jop.2009.080007. PMID: 19186958; PMCID: PMC2778200. 9. Chung WC, Kao CC, Huang CF, Lee CY, Lu HK, Wu MS. Effects of Periodontal Treatment in Patients with Periodontitis and Kidney Failure: A Pilot Study. Int J Environ Res Public Health. 2022 Jan 29;19(3):1533. doi: 10.3390/ijerph19031533 Add to Citavi project by DOI. PMID: 35162556 Add to Citavi project by Pubmed ID; PMCID: PMC8835327. 10. Rapone B, Ferrara E, Corsalini M, Qorri E, Converti I, Lorusso F, Delvecchio M, Gnoni A, Scacco S, Scarano A. Inflammatory Status and Glycemic Control Level of Patients with Type 2 Diabetes and Periodontitis: A Randomized Clinical Trial. Int J Environ Res Public Health. 2021 Mar 15;18(6):3018. doi: 10.3390/ijerph18063018 Add to Citavi project by DOI. PMID: 33804123 Add to Citavi project by Pubmed ID; PMCID: PMC7998112. 11. de Pablo P, Serban S, Lopez-Oliva I, Rooney J, Hill K, Raza K, Filer A, Chapple I, Dietrich T. Outcomes of periodontal therapy in rheumatoid arthritis: The OPERA feasibility randomized trial. J Clin Periodontol. 2023 Mar;50(3):295-306. doi: 10.1111/jcpe.13756. Epub 2022 Dec 16. PMID: 36415901. 12. Brinar S, Skvarča A, Gašpirc B, Schara R. The effect of antimicrobial photodynamic therapy on periodontal disease and glycemic control in patients with type 2 diabetes mellitus. Clin Oral Investig. 2023 Oct;27(10):6235-6244. doi: 10.1007/s00784-023-05239-0 Add to Citavi project by DOI. Epub 2023 Sep 6. PMID: 37672083 Add to Citavi project by Pubmed ID; PMCID: PMC10560165. 13. Liu F, Sui W, Zhou ZF, Mi Y, He TQ, Li ZB, Hong YL, Chen FM. Development of gestational diabetes mellitus in women with periodontitis in early pregnancy: A population-based clinical study. J Clin Periodontol. 2022 Feb;49(2):164-176. doi: 10.1111/jcpe.13578 Add to Citavi project by DOI. Epub 2021 Dec 14. PMID: 34865247. 14. Čolak D, Cmok Kučič A, Pintar T, Gašperšič R. Periodontal Therapy in Bariatric Surgery Patients with Periodontitis: Randomized Control Clinical Trial. J Clin Med. 2022 Nov 19;11(22):6837. doi: 10.3390/jcm11226837. PMID: 36431314; PMCID: PMC9693218. 15. Geisinger ML, Michalowicz BS, Hou W, Schoenfeld E, Gelato M, Engebretson SP, Reddy MS, Hyman L. Systemic Inflammatory Biomarkers and Their Association With Periodontal and Diabetes-Related Factors in the Diabetes and Periodontal Therapy Trial, A Randomized Controlled Trial. J Periodontol. 2016 Aug;87(8):900-13. doi: 10.1902/jop.2016.150727. Epub 2016 Apr 25. PMID: 27108476. | Miscellaneous reasons (n=15) |

**Appendix Table 3:** Baseline, 3 and 6 months mean ± standard deviations of mean probing depth, mean clinical attachment loss, percentage of sites with bleeding on probing, and percentage of sites with probing depths ≤3 mm.

| **Study** | **Gr** | **Mean PD, mm** | | | **Mean CAL, mm** | | | **%BOP, %** | | | **%PD≤3mm, %** | | |
| --- | --- | --- | --- | --- | --- | --- | --- | --- | --- | --- | --- | --- | --- |
|  |  | **Baseline** | **3 m** | **6 m** | **Baseline** | **3 m** | **6 m** | **Baseline** | **3 m** | **6 m** | **Baseline** | **3 m** | **6 m*** |
| Adegboye 2021 | I | 4.24±0.23 |  | 4.22±0.26 | 4.21±0.21 |  | 4.29±0.34 | 0.20±0.14 |  | 0.09±0.07 |  |  |  |
|  | C | 4.3±0.27 |  | 4.29±0.17 | 4.28±0.24 |  | 4.29±0.17 | 0.23±0.16 |  | 0.29±0.18 |  |  |  |
| Akram 2017 | I | 2.33±0.44 | 1.96±0.47 |  | 2.89±0.61 | 2.45±0.59 |  |  |  |  | 90.70±9.76 | 96.34±7.18 |  |
|  | C | 2.40±0.37 | 2.11±0.41 |  | 3.06±0.66 | 2.63±0.67 |  |  |  |  | 87.41±9.97 | 93.08±8.85 |  |
| Caneiro-Queija 2019 | I |  |  |  | 0.77±0.46 | 0.69±0.57 |  | 54.57±18.61 | 27.88±15.29 |  | 52.42±21.43 | 70.35±17.46 |  |
|  | C |  |  |  | 0.50±0.32 | 0.60±0.35 |  | 42.59±18.00 | 43.21±20.77 |  | 70.00±14.54 | 65.22±16.81 |  |
| Chen 2012 | I | 2.57±0.66 | 2.2±0.39 | 2.10±0.39 | 2.95±1.21 | 2.55±1.15 | 2.55±1.16 | 32.42±16.63 | 12.13±8.24 | 12.02±8.99 |  |  |  |
|  | C | 2.47±0.57 | 2.38±0.47 | 2.42±0.50 | 3.37±1.24 | 3.29±1.23 | 3.41±1.23 | 34.01±18.91 | 28.53±14.42 | 28.37±13.5 |  |  |  |
| Das 2019 | I | 3.08±0.30 | 2.32±0.28 |  | 3.79±0.40 | 3.06±0.42 |  |  |  |  |  |  |  |
|  | C | 3.42±0.49 | 3.52±0.49 |  | 3.84±0.50 | 3.91±0.49 |  |  |  |  |  |  |  |
| Deepti 2017 | I | 2.63±0.25 |  | 1.16±0.37 | 1.33±0.69 |  | 0.34±0.16 | 90.05±7.77 |  | 30.48±16.53 |  |  |  |
|  | C | 2.51±0.29 |  | 2.60±0.41 | 1.26±0.70 |  | 1.34±0.74 | 87.18±9.20 |  | 84.85±15.33 |  |  |  |
| El-Makaky 2020 | I | 4.78±0.65 | 4.25±0.62 |  | 4.74±0.60 | 4.31±0.59 |  | 48.56±27.38 | 6.20±7.14 |  |  |  |  |
|  | C | 4.79±0.63 | 4.94±0.65 |  | 4.74±0.66 | 4.96±0.63 |  | 48.47±31.23 | 49.03±29.98 |  |  |  |  |
| Eltas 2013 | I | 3.62±0.64 | 2.77±0.59 |  | 4.14±0.76 | 3.45±0.67 |  | 68.00±15.00 | 28.00±5.00 |  |  |  |  |
|  | C | 3.88±0.58 | 3.79±0.51 |  | 4.20±0.85 | 4.20±0.84 |  | 67.00±16.00 | 63.00±15.00 |  |  |  |  |
| Engebretson 2013 | I | 3.26±0.60 | 2.82±0.53 | 2.78±0.39 | 3.48±0.80 | 3.16±0.78 | 3.12±0.71 | 61.20±24.10 | 40.00±23.32 |  |  |  |  |
|  | C | 3.28±0.70 | 3.15±0.60 | 3.14±0.76 | 3.49±0.90 | 3.42±0.92 | 3.44±0.97 | 59.60±26.00 | 57.00±23.25 |  |  |  |  |
| Fang 2015 | I | 3.09±0.46 | 2.55±0.43 | 2.46±0.33 |  |  |  | 43.59±15.06 | 18.13±10.75 | 16.28±9.12 | 79.99±8.02 | 88.47±5.09 | 89.02±3.51 |
|  | C | 3.06±0.61 | 2.92±0.46 | 2.98±0.50 |  |  |  | 42.95±14.81 | 31.20±9.11 | 32.15±10.92 | 81.52±5.91 | 84.02±6.56 | 83.21±5.22 |
| Fiorini 2013 | I |  |  |  |  |  |  | 49.62±20.74 |  | 12.29±8.04 |  |  |  |
|  | C |  |  |  |  |  |  | 45.65±17.52 |  | 30.32±17.17 |  |  |  |
| Hada 2015 | I | 1.98±0.48 |  | 1.78±0.54 | 2.92±0.63 |  | 2.85±0.59 | 64.83±21.22 |  | 38.97±12.93 |  |  |  |
|  | C | 1.79±0.38 |  | 1.86±0.31 | 2.67±0.72 |  | 2.78±0.64 | 85.96±12.93 |  | 83.2±13.02 |  |  |  |
| Ide 2003 | I |  |  |  |  |  |  | 41.75±17.44 | 15.63±10.37 |  | 55.24±17.98 | 71.62±15.33 |  |
|  | C |  |  |  |  |  |  | 45.36±23.46 | 39.45±18.85 |  | 55.11±17.43 | 58.78±17.88 |  |
| Kamil 2011 | I |  |  |  |  |  |  |  |  |  | 60.80±10.80 | 95.40±3.60 |  |
|  | C |  |  |  |  |  |  |  |  |  | 59.00±10.80 | 58.80±10.80 |  |
| Kapellas 2014 | I | 2.39±0.52 | 2.23±0.47 |  |  |  |  |  |  |  |  |  |  |
|  | C | 2.41±0.59 | 2.36±0.53 |  |  |  |  |  |  |  |  |  |  |
| Kaur 2015 | I | 2.96±0.46 | 2.17±0.43 | 2.15±0.42 | 3.46±0.53 | 2.77±0.62 | 2.75±0.62 | 73.68±14.63 | 39.07±11.68 | 38.96±11.62 | 37.46±8.67 | 58.94±12.32 | 58.94±12.25 |
|  | C | 3.08±0.55 | 3.10±0.56 | 3.13±0.57 | 3.37±0.61 | 3.40±0.62 | 3.44±0.64 | 75.36±10.49 | 76.99±11.26 | 78.88±11.84 | 35.97±9.67 | 33.84±10.29 | 31.82±11.44 |
| Kiran 2005 | I | 2.29±0.49 | 1.80±0.25 |  | 3.19±1.13 | 2.80±1.03 |  | 54.38±18.75 | 23.90±12.73 |  |  |  |  |
|  | C | 2.24±0.70 | 2.26±0.63 |  | 2.92±1.10 | 2.87±1.03 |  | 50.48±26.10 | 51.91±27.38 |  |  |  |  |
| Kolte 2022 | I | 6.59±1.50 | 5.17±1.46 |  | 7.20±1.60 | 5.56±1.39 |  | 64.10±3.58 | 63.19±1.27 |  |  |  |  |
|  | C | 6.13±0.83 | 6.14±0.83 |  | 6.52±0.78 | 6.52±0.77 |  | 64.10±7.67 | 64.02±7.47 |  |  |  |  |
| Koromantzos 2011 | I |  |  |  |  |  |  | 71.55±27.00 |  | 33.43±27.00 |  |  |  |
|  | C |  |  |  |  |  |  | 69.27±25.9 |  | 64.92±25.90 |  |  |  |
| López 2002 | I | 2.71±0.30 | 2.10±0.30 |  | 1.86±0.62 | 1.04±0.68 |  | 49.90±16.20 | 14.90±2.40 |  |  |  |  |
|  | C | 2.94±0.42 | 2.98±0.40 |  | 1.75±0.73 | 1.84±0.67 |  | 55.40±19.80 | 62.50±14.00 |  |  |  |  |
| Masi 2018 | I | 3.90±0.80 |  | 2.90±0.70 |  |  |  | 70.00±20.00 |  | 36.00±21.00 |  |  |  |
|  | C | 3.60±0.70 |  | 3.30±0.70 |  |  |  | 72.00±15.00 |  | 58.00±18.00 |  |  |  |
| Milanesi 2022 | I | 3.02±0.71 | 2.33±0.35 | 2.27±0.35 | 3.96±1.42 | 3.49±1.24 | 3.39±1.24 | 58.00±24.44 | 22.40±13.50 | 19.60±10.22 |  |  |  |
|  | C | 2.95±0.79 | 2.80±0.71 | 2.79±0.79 | 4.04±1.51 | 3.89±1.51 | 3.87±1.51 | 58.70±25.40 | 51.90±24.61 | 50.70±26.39 |  |  |  |
| Mizuno 2017 | I | 2.40±0.50 | 2.10±0.50 | 2.10±0.50 | 2.60±0.60 | 2.30±0.50 | 2.40±0.50 | 29.40±21.40 | 22.40±22.40 | 20.80±25.00 |  |  |  |
|  | C | 2.40±0.70 | 2.30±0.70 | 2.30±0.50 | 2.70±0.90 | 2.60±0.90 | 2.50±0.40 | 23.10±17.20 | 25.10±17.10 | 26.10±20.40 |  |  |  |
| Moeintaghavi 2012 | I | 2.31±0.65 | 2.21±0.60 |  | 3.14±1.08 | 2.80±1.09 |  |  |  |  |  |  |  |
|  | C | 2.06±0.24 | 2.33±0.30 |  | 3.10±1.05 | 3.47±1.44 |  |  |  |  |  |  |  |
| Montenegro 2019 | I | 3.33±0.57 | 2.43±0.51 |  | 5.29±1.40 | 4.51±1.19 |  | 93.90±9.30 | 30.20±32.10 |  |  |  |  |
|  | C | 3.11±0.52 | 3.27±0.75 |  | 4.90±1.29 | 5.04±1.35 |  | 88.70±110 | 74.70±20 |  |  |  |  |
| Offenbacher 2006 | I | 2.28±0.07 | 1.46±0.07 |  | 0.61±0.06 | 0.45±0.04 |  | 43.90±6.20 | 11.50±3.90 |  |  |  |  |
|  | C | 2.01±0.06 | 2.39±0.07 |  | 0.56±0.06 | 0.58±0.03 |  | 47.70±4.90 | 39.50±3.70 |  |  |  |  |
| Offenbacher 2009 | I | 2.69±0.06 |  | 2.41±0.06 | 2.79±0.08 |  | 2.52±0.08 | 48.00±3.13 |  | 38.30±2.17 |  |  |  |
|  | C | 2.72±0.05 |  | 2.57±0.07 | 2.95±0.09 |  | 2.72±0.11 | 47.30±2.18 |  | 42.50±2.56 |  |  |  |
| Pham 2022 | I | 3.40±0.30 | 3.00±0.70 | 2.80±0.60 | 3.70±0.90 | 3.10±0.60 | 2.90±0.70 | 63.40±22.40 | 20.60±12.40 | 18.10±8.60 |  |  |  |
|  | C | 3.40±0.20 | 3.40±0.30 | 3.50±0.20 | 3.70±0.50 | 3.80±0.50 | 3.80±0.50 | 63.30±9.40 | 44.70±19.50 | 55.20±14.20 |  |  |  |
| Pinho 2009 | I | 3.24±0.88 | 2.82±0.77 | 2.71±0.76 |  |  |  | 49.47±35.19 | 25.91±28.93 | 29.78±28.93 |  |  |  |
|  | C | 3.12±0.47 | 3.12±0.47 | 3.13±0.45 |  |  |  | 34.25±18.53 | 34.25±18.53 | 34.25±18.53 |  |  |  |
| Qureshi 2021 | I | 3.44±0.95 | 2.68±0.82 |  | 3.74±0.94 | 2.54±0.76 |  | 25.22±8.63 | 11.66±4.95 |  |  |  |  |
|  | C | 3.04±0.73 | 3.43±0.95 |  | 3.55±1.11 | 3.60±0.91 |  | 22.08±8.02 | 23.61±12.82 |  |  |  |  |
| Raman 2014 | I | 2.56±0.57 | 1.76±0.19 |  | 3.35±0.83 | 2.73±0.70 |  |  |  |  | 81.14±13.38 | 97.96±1.75 |  |
|  | C | 2.29±0.69 | 2.02±0.71 |  | 2.79±0.96 | 2.56±0.97 |  |  |  |  | 87.54±15.00 | 91.67±14.86 |  |
| Ribeiro 2005 | I |  |  |  |  |  |  | 51.40±27.90 | 8.50±5.80 |  | 71.90±14.40 | 90.00±7.30 |  |
|  | C |  |  |  |  |  |  | 58.00±28.80 | 37.40±26.60 |  | 70.80±20.70 | 74.80±18.40 |  |
| Sadatmansouri 2006 | I | 2.30±0.30 | 2.10±0.30 |  | 2.30±0.30 | 2.00±0.30 |  | 16.60±7.20 | 0.70±4.20 |  |  |  |  |
|  | C | 2.30±0.40 | 2.50±0.50 |  | 2.20±0.30 | 2.30±0.40 |  | 13.40±6.40 | 17.20±3.30 |  |  |  |  |
| Saffi 2018 | I | 3.22±0.54 | 2.27±0.51 |  | 5.12±1.46 | 4.31±1.26 |  | 92.49±10.10 | 34.08±33.32 |  |  |  |  |
|  | C | 3.07±0.54 | 3.16±0.73 |  | 4.89±1.33 | 4.91±1.35 |  | 88.21±11.56 | 71.74±21.39 |  |  |  |  |
| Seinost 2006 | I | 3.30±0.59 | 3.10±0.59 |  | 4.16±0.95 | 4.04±0.95 |  | 62.90±16.86 | 35.22±16.86 |  |  |  |  |
|  | C | 3.53±1.16 | 3.71±1.16 |  | 4.54±1.76 | 4.77±1.76 |  | 64.79±20.64 | 51.83±20.64 |  |  |  |  |
| Singh 2008 | I | 2.67±0.35 | 2.33±0.35 |  | 3.44±0.45 | 3.14±0.45 |  |  |  |  |  |  |  |
|  | C | 2.44±0.26 | 2.40±0.46 |  | 2.78±0.33 | 2.83±0.35 |  |  |  |  |  |  |  |
| Tran 2021 | I | 1.21±0.27 | 1.13±0.13 | 1.06±01.17 | 1.96±0.40 | 1.81±0.39 | 1.78±0.37 |  |  |  |  |  |  |
|  | C | 1.20±0.15 | 1.20±0.12 | 1.21±0.15 | 1.97±0.40 | 2.06±0.33 | 2.20±0.36 |  |  |  |  |  |  |
| Telgi 2013 | I | 5.05±0.70 | 4.59±0.72 |  |  |  |  |  |  |  |  |  |  |
|  | C | 5.05±0.69 | 5.03±0.69 |  |  |  |  |  |  |  |  |  |  |
| Vidal 2009 | I |  |  |  |  |  |  | 45.70±18.20 | 19.40±11.40 |  |  |  |  |
|  | C |  |  |  |  |  |  | 42.70±15.10 | 40.30±12.20 |  |  |  |  |
| Wang S 2017 | I | 3.66±0.60 | 3.09±0.63 |  | 4.12±0.90 | 3.62±0.84 |  |  |  |  |  |  |  |
|  | C | 3.85±0.58 | 3.92±0.56 |  | 4.28±0.77 | 4.36±0.81 |  |  |  |  |  |  |  |
| Wang Y 2017 | I |  |  |  |  |  |  | 54.60±21.50 |  | 16±9.6 |  |  |  |
|  | C |  |  |  |  |  |  | 57.50±24.90 |  | 66.4±39.3 |  |  |  |
| Wang Y 2020 | I | 3.10±1.21 |  | 2.37±0.57 | 4.29±1.77 |  | 3.89±1.11 | 56.00±20.10 |  | 27.60±19.60 |  |  |  |
|  | C | 2.92±0.99 |  | 3.01±0.74 | 3.97±1.32 |  | 4.28±1.35 | 55.40±26.10 |  | 55.62±29.44 |  |  |  |
| Wu 2015 | I | 3.73±0.58 | 3.01±0.63 | 3.10±0.62 | 3.66±1.29 | 3.18±1.26 | 3.24±1.16 |  |  |  |  |  |  |
|  | C | 3.71±0.61 | 3.47±0.67 | 3.51±0.71 | 3.90±1.18 | 3.96±1.18 | 4.00±1.17 |  |  |  |  |  |  |
| Zhou S 2013 | I | 3.95±0.13 | 2.94±0.20 |  | 3.07±0.46 | 2.45±0.47 |  |  |  |  |  |  |  |
|  | C | 3.92±0.23 | 3.93±0.23 |  | 2.97±0.46 | 2.99±0.45 |  |  |  |  |  |  |  |
| Zhou X 2014 | I | 3.13±0.55 |  | 2.56±0.35 | 4.58±1.72 |  | 4.08±1.41 |  |  |  |  |  |  |
|  | C | 3.12±0.62 |  | 3.16±0.62 | 5.27±1.69 |  | 5.50±1.65 |  |  |  |  |  |  |

PD: Probing depth; CAL: Clinical attachment loss; %BOP: Percentage of sites with bleeding on probing; %PD≤3mm: Percentage of sites with probing depth ≤3 mm; Gr: Group; I: Intervention group; C: Control group

**Appendix Table 4:** Results from the random-effects meta-regression models for all outcomes assessed at baseline and 3 months regressed over systemic diseases, risk of bias (high vs. low/ some concerns) and year of publication.

|  | **Mean PD, mm** | **Mean CAL, mm** | **%BOP, %** | **%PD≤3mm, %** |
| --- | --- | --- | --- | --- |
| **R^2^**  **(Accounted heterogeneity)** | 38.65% | 50.89% | 36.86% | 63.52% |
| **Estimate (95% C.I.)** | | | | |
| **Intercept** | 31.057  (-22.579; 84.693) | 15.868  (-32.714; 64.451) | -696.042  (-2444.69; 1052.61) | -1307.944  (-3281.17; 665.28) |
| **CVD** | Ref. | Ref. | Ref. | Ref. |
| **DM** | 0.302  (-0.013; 0.618) | 0.325  (-0.059; 0.710) | 10.321  (-3.101; 23.744) | -13.748*  (-26.211; -1.286) |
| **ED** | -0.277  (-0.787; 0.232) | -0.113  (-0.640; 0.415) | -11.937  (-30.919; 7.044) | -7.732  (-24.938; 9.473) |
| **Healthy** | - | - | - | 7.944  (-10.108; 25.995) |
| **Obesity** | 0.438  (-0.196; 1.071) | 0.406  (-0.251; 1.063) | - | -22.590*  (-39.689; -5.492) |
| **Pregnancy** | -0.242  (-0.752; 0.268) | 0.259  (-0.210; 0.729) | 2.442  (-13.003; 17.888) | -6.138  (-20.229; 7.953) |
| **RA** | 0.162  (-0.630; 0.954) | - | 8.769  (-12.614; 30.152) | 10.820  (-8.876; 30.515) |
| **CKD** | 0.458  (-0.151; 1.068) | - | 18.259  (-4.725; 41.243) | -26.825*  (-45.239; -8.410) |
| **RoB, High** | Ref. | Ref. | Ref. | Ref. |
| **RoB, Low** | 0.100  (-0.248; 0.447) | -0.096  (-0.474; 0.282) | -2.688  (-17.289; 11.913) | 13.616  (-1.556; 28.787) |
| **RoB, Some concerns** | -0.173  (-0.471; 0.156) | -0.410*  (-0.742; -0.077) | -6.776  (-21.662; 8.111) | 20.349*  (4.608; 36.091) |
| **Year** | -0.016  (-0.042; 0.011) | -0.008  (-0.032; 0.016) | 0.333  (-0.535; 1.202) | 0.655  (-0.326; 1.635) |

PD: Probing depth; CAL: Clinical attachment loss; %BOP: Percentage of sites with bleeding on probing; %PD≤3mm: Percentage of sites with probing depth ≤3 mm; CVD: Cardiovascular diseases; DM: Diabetes mellitus; ED: Erectile dysfunction; RA: Rheumatoid Arthritis; CKD: Chronic kidney disease; RoB: Risk of bias.

* Indicate statistically significant associations (p < 0.05)

**Appendix Table 5.** Results from the random-effects meta-regression models for all outcomes assessed at baseline and 6 months regressed over systemic diseases, risk of bias (high vs. low/ some concerns) and year of publication.

|  | **Mean PD, mm** | **Mean CAL, mm** | **%BOP, %** |
| --- | --- | --- | --- |
| **R^2^**  **(Accounted heterogeneity)** | 73.55% | 91.09% | 81.53% |
| **Estimate (95% C.I.)** | | | |
| **Intercept** | 2.681  (-68.987; 73.350) | 20.583  (-46.273; 87.439) | 2263.273  (-50.398; 4576.944) |
| **COPD** | Ref. | Ref. | Ref. |
| **CVD** | 0.613  (-0.056; 1.283) | 1.647*  (0.569; 2.725) | - |
| **DM** | 0.192  (-0.322; 0.706) | 1.099*  (0.094; 2.104) | 15.614*  (0.739; 30.489) |
| **PCOS** | -0.743*  (-1.411; -0.076) | 0.599  (-0.469; 1.667) | -7.857  (-26.851; 11.138) |
| **Pregnancy** | 0.541  (-0.097; 1.179) | 1.498*  (0.465; 2.531) | 16.929  (-3.806; 37.664) |
| **RA** | 0.172  (-0.572; 0.915) | - | 25.922  (-1.143; 52.987) |
| **CKD** | 0.173  (-0.478; 0.825) | - | 28.360*  (10.844; 45.876) |
| **RoB, High** | Ref. | Ref. | - |
| **RoB, Low** | 0.000  (-0.554; 0.554) | 0.428  (-0.315; 1.170) | Ref.^a^ |
| **RoB, Some concerns** | -0.092  (-0.643; 0.459) | 0.282  (-0.436; 0.999) | -6.988  (-16.248; 2.272) |
| **Year** | -0.002  (-0.037; 0.034) | -0.011  (-0.044; 0.022) | -1.142  (-2.290; 0.006) |

PD: Probing depth; CAL: Clinical attachment loss; %BOP: Percentage of sites with bleeding on probing

* Indicate statistically significant associations (p < 0.05)

^a^ All studies included in this meta-regression had either low or ‘some concerns’ risk of bias scores. Therefore, low RoB was considered as the reference category and the estimates were produced for ‘some concerns’ group.

**Appendix Figure 1.** Flow chart depicting the results of the search, the selection process, and the number of studies included in this systematic review.


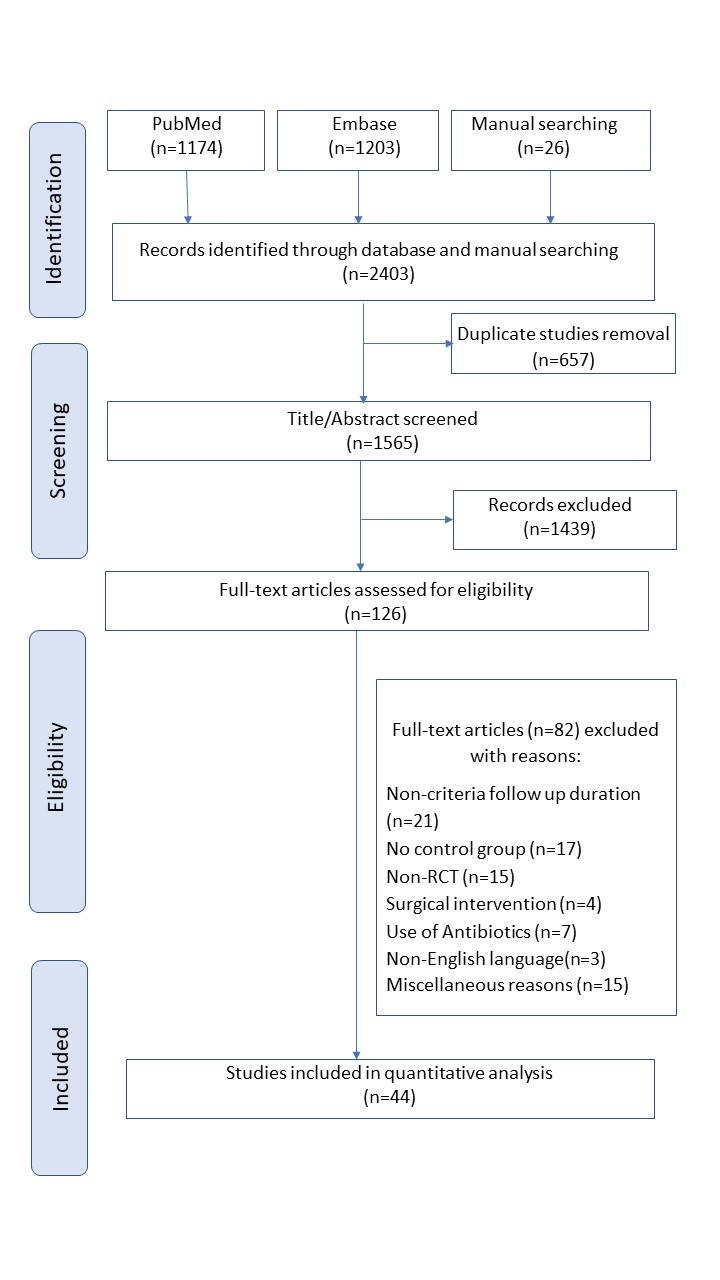


**Appendix Figure 2:** Forest plot showing the mean differences between baseline and follow-up examinations (at 3 or 6 months) for all outcome variables in the treatment arm.


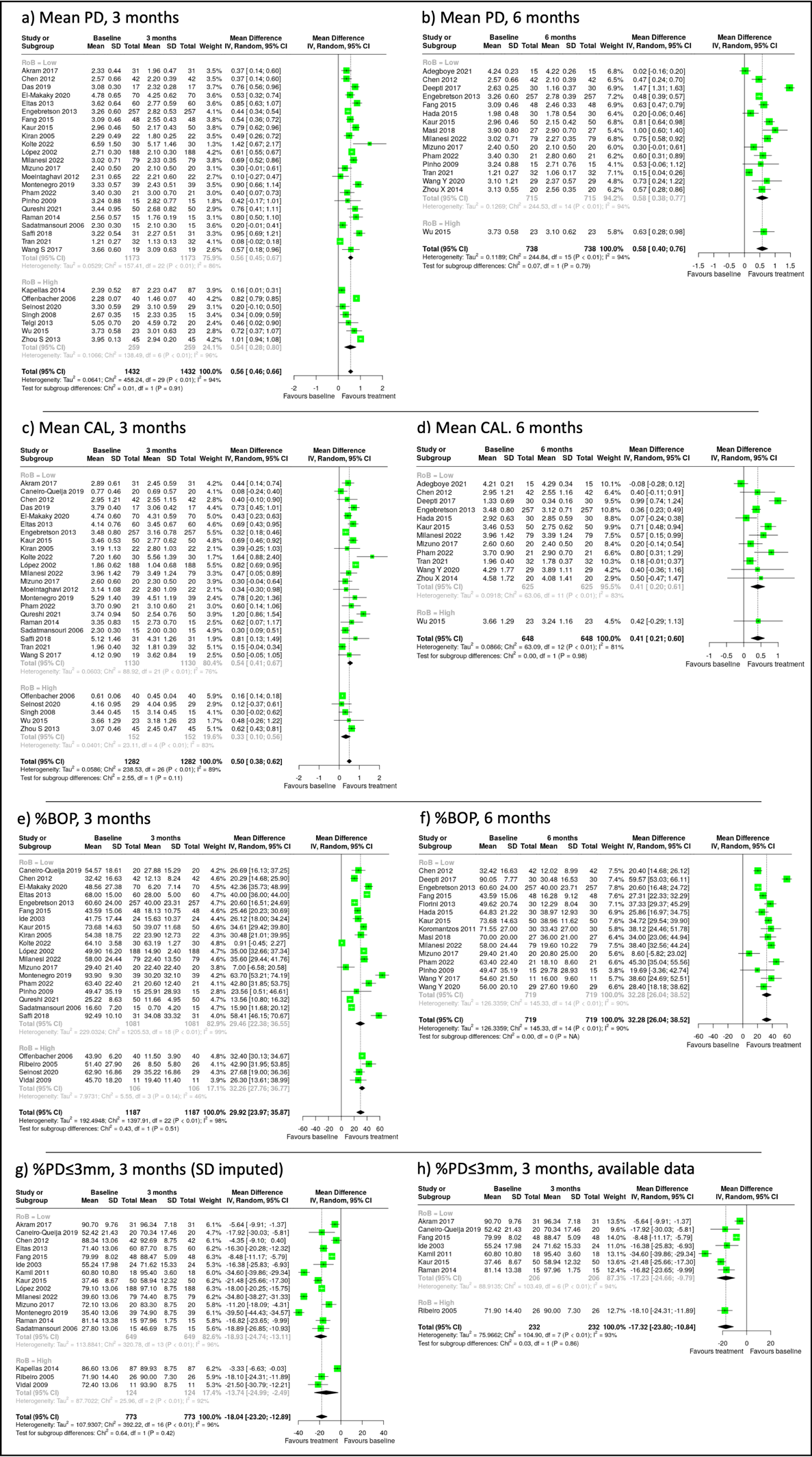

Supplement: Supplementary file 1 — Supplementary file1 (DOCX 1217 KB) [file 784_2023_5392_MOESM1_ESM.docx]
